# Air-liquid interface exposure to aerosols of poorly soluble nanomaterials induces different biological activation levels compared to exposure to suspensions

**DOI:** 10.1186/s12989-016-0171-3

**Published:** 2016-11-03

**Authors:** Thomas Loret, Emmanuel Peyret, Marielle Dubreuil, Olivier Aguerre-Chariol, Christophe Bressot, Olivier le Bihan, Tanguy Amodeo, Bénédicte Trouiller, Anne Braun, Christophe Egles, Ghislaine Lacroix

**Affiliations:** 1 Institut National de l’Environnement Industriel et des Risques (INERIS), (DRC/VIVA/TOXI), Parc Technologique ALATA—BP 2, Verneuil-en-Halatte, F-60550 France; 2 Laboratoire BioMécanique et BioIngénierie (BMBI), Université de Technologie de Compiègne (UTC), UMR CNRS 7338, Compiègne, 60205 France; 3 Institut National de l’Environnement Industriel et des Risques (INERIS), (DRC/CARA/NOVA), Parc Technologique ALATA—BP 2, Verneuil-en-Halatte, F-60550 France; 4 Department of Biomedical Engineering, Tufts University, Medford, MA USA

**Keywords:** Nanomaterials, In vitro, Alveolar cells, Co-culture, Air-liquid interface, Submerged conditions, Toxicity

## Abstract

**Background:**

Recently, much progress has been made to develop more physiologic in vitro models of the respiratory system and improve in vitro simulation of particle exposure through inhalation. Nevertheless, the field of nanotoxicology still suffers from a lack of relevant in vitro models and exposure methods to predict accurately the effects observed in vivo, especially after respiratory exposure. In this context, the aim of our study was to evaluate if exposing pulmonary cells at the air-liquid interface to aerosols of inhalable and poorly soluble nanomaterials generates different toxicity patterns and/or biological activation levels compared to classic submerged exposures to suspensions. Three nano-TiO_2_ and one nano-CeO_2_ were used. An exposure system was set up using VitroCell® devices to expose pulmonary cells at the air-liquid interface to aerosols. A549 alveolar cells in monocultures or in co-cultures with THP-1 macrophages were exposed to aerosols in inserts or to suspensions in inserts and in plates. Submerged exposures in inserts were performed, using similar culture conditions and exposure kinetics to the air-liquid interface, to provide accurate comparisons between the methods. Exposure in plates using classical culture and exposure conditions was performed to provide comparable results with classical submerged exposure studies. The biological activity of the cells (inflammation, cell viability, oxidative stress) was assessed at 24 h and comparisons of the nanomaterial toxicities between exposure methods were performed.

**Results:**

Deposited doses of nanomaterials achieved using our aerosol exposure system were sufficient to observe adverse effects. Co-cultures were more sensitive than monocultures and biological responses were usually observed at lower doses at the air-liquid interface than in submerged conditions. Nevertheless, the general ranking of the nanomaterials according to their toxicity was similar across the different exposure methods used.

**Conclusions:**

We showed that exposure of cells at the air-liquid interface represents a valid and sensitive method to assess the toxicity of several poorly soluble nanomaterials. We underlined the importance of the cellular model used and offer the possibility to deal with low deposition doses by using more sensitive and physiologic cellular models. This brings perspectives towards the use of relevant in vitro methods of exposure to assess nanomaterial toxicity.

**Electronic supplementary material:**

The online version of this article (doi:10.1186/s12989-016-0171-3) contains supplementary material, which is available to authorized users.

## Background

The growing utilization of nanomaterials (NMs) in nanotechnologies leads to an increased risk of human exposure [1], raising concerns about public health and safety [2–4]. Metallic and poorly soluble NMs are among the most widely used [5] and a major exposure route for these NMs is inhalation [6]. Nevertheless, occupational and environmental atmospheres have not been well characterized in terms of NMs [7], which partly explains the lack of epidemiological data on the relationship between exposure to airborne NMs and potential adverse human health effects. However, based on epidemiological studies showing an association between exposure to environmental ultrafine particles and adverse health effects [8], the potential toxicity of NM has been taken into consideration and been widely studied in cell cultures and animal models [9, 10]. Results from animal experimentations remain the most reliable [11, 12], especially because of the similar level of complexity compared with the human body. Besides ethical considerations, in vitro studies are widely used to study mechanisms of toxicity because they are usually cheaper, faster and easier to implement than in vivo studies [13]. Nevertheless, the relevance of in vitro studies to predict in vivo effects needs to be carefully assessed.

In vivo, inhaled NMs can deposit in the alveolar region [14, 15] and interact with components of the alveolar barrier at the air-liquid interface (ALI) [15]. At the apical side of the barrier, insoluble NMs first interact with the thin layer of surfactant secreted by pneumocytes [16]. This layer covers the entire alveolar surface and transport of NMs occurs from the air to the aqueous surfactant phase [15]. NMs can then be taken up by circulating macrophages to be eliminated or interact directly with pneumocytes [15, 17]. If NMs cross the alveolar barrier [18, 19], they can interact with other components of the barrier such as endothelial cells or immune cells and be transferred to the blood and other organs [19, 20]. As a consequence of the particle-cell interactions, mechanisms of defense can become activated and cell damages can occur such as cell function impairment, release of pro- and anti-inflammatory cytokines, production of intracellular Reactive Oxygen Species (ROS) and anti-oxidant species, and genotoxicity [4, 6, 21].

In vitro, monocultures of pulmonary cells are usually exposed in submerged conditions to suspensions of NMs to determine mechanisms of toxicity [21] or high throughput screening of novel compounds [13]. However, these experimental conditions do not reflect cell-cell communications and cell-particle interactions occurring in vivo in the lung, making in vitro results difficult to interpret [11, 12, 22–24]. Moreover, in submerged conditions, cell-particle interactions are dependent on the medium composition [25, 26]. NMs can interact with components of the culture medium, resulting in the formation of a medium specific corona [26, 27] and can agglomerate into larger particles of different sizes. Furthermore, in suspensions the dose delivered to the cells depends on the NM properties in suspension and capacity to settle, which makes in vitro dosimetry complex [28–30].

To overcome these difficulties, more complex cellular models [31, 32] and new in vitro exposure methods [23, 33] have been and are still being developed to study NM toxicity. Co-culture models are used to mimic communications occurring between different cell types in vivo in the lungs [9, 22]. These models associate various cells such as epithelial, macrophage, endothelial or dendritic cells [31]. In vitro systems exposing cells to aerosols of NMs at the ALI have been developed to accurately mimic the cell-particle interactions occurring in the lungs [23]. With these ALI systems, NM deposition on cells occurs through diffusion and/or gravitational mechanisms [23]. However, due to current technical limitations, the maximum doses achieved in these systems remain generally low compared to those achievable through suspension exposure. More recently, in order to improve the deposition rate, exposure devices using electrostatic deposition of charged particles [23, 34] or thermal precipitation [35] have been introduced. However, it has not yet been clearly defined whether in vitro simulation of in vivo exposure conditions to test NM toxicity gives more predictive results.

As a first step to address this question, the aim of our study was to assess whether ALI exposures to NM aerosols give similar results in terms of cellular responses, compared to submerged exposures to NM suspensions. For this purpose, we set up an in vitro system allowing exposure of cell cultures to aerosols of poorly-soluble metallic NMs. We exposed two alveolar models in inserts: monocultures of alveolar epithelial cells A549 and co-cultures of A549 and macrophage like cells THP-1 at the ALI to aerosols of three TiO_2_ and one CeO_2_ NM. In parallel, we performed submerged exposures in inserts to suspensions of the NMs, using similar culture conditions and exposure kinetics. We also performed classical culture and exposure conditions in plates. We assessed the biological activity of the cells (release of pro-inflammatory markers, cell functionality, cell integrity, intracellular reactive oxygen species (ROS) levels) after 24 h of exposure and compared the toxicities of the NMs between aerosol and suspension exposure.

## Results and Discussion

**Fig. 1:**
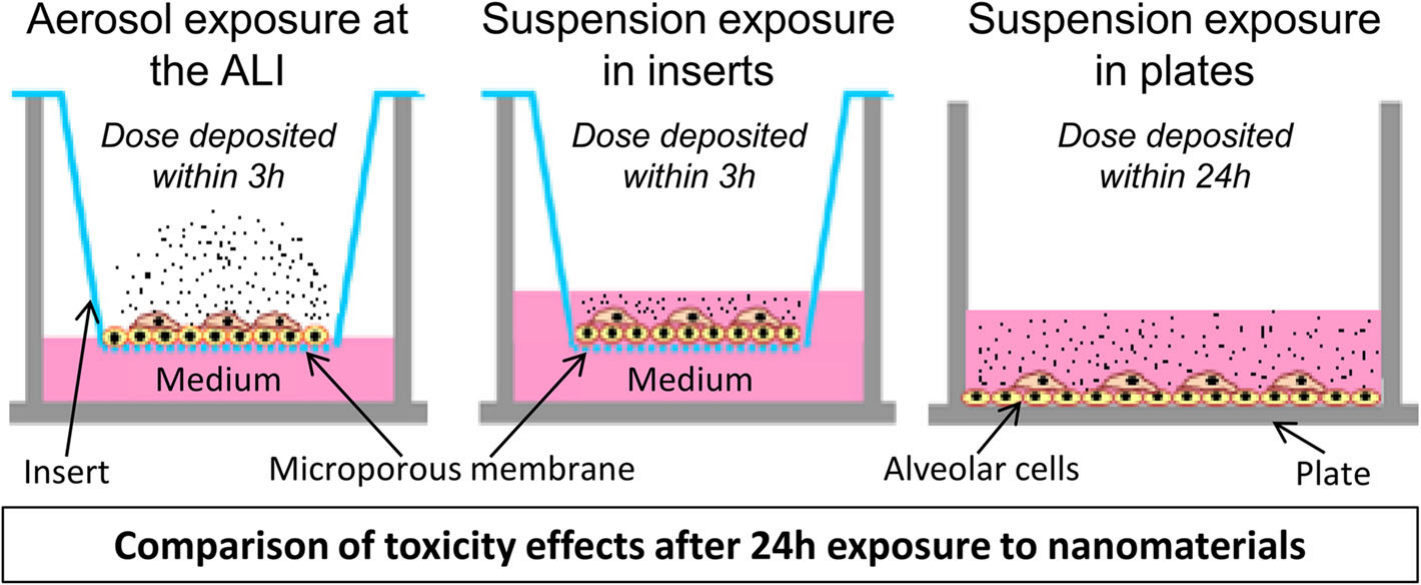
In vitro comparisons between ALI and submerged exposure. Alveolar cells in monoculture or in co-culture were cultured in inserts or in plates and exposed at the ALI to aerosols or in submerged conditions to suspensions of four poorly soluble NMs. Final doses were reached within 3 h in inserts and 24 h in plates. Total deposited doses were measured at the ALI or estimated in submerged conditions and cell biological activity was assessed after 24 h of exposure to the NMs, performing cell viability, stress oxidative and inflammation assays. Comparisons were performed between the biological activation levels determined after statistical analysis

The aim of our study was to evaluate if exposing alveolar cells at the ALI to aerosols of inhalable and poorly soluble NMs would generate different toxicity patterns and/or biological activation levels compared to submerged exposures to suspensions (Fig. 1).

### Exposures at the air-liquid interface to aerosols of NM

**Fig. 2:**
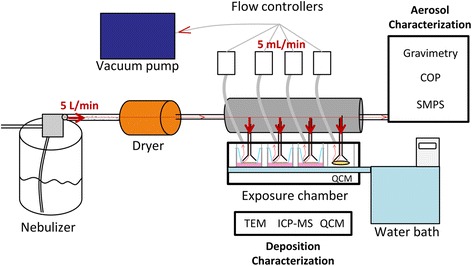
Exposure of cells at the ALI to aerosols of NMs. Cells at the ALI in inserts were exposed simultaneously to aerosols of NMs or to air in two different VitroCell® exposure chambers. NM aerosols were generated at a 5 L/min flow rate by nebulization of suspensions using a nebulizer. Aerosols were dried using a dryer to reduce relative humidity to 90 %. The aerosols were sucked using a vacuum pump to allow the NM deposition on the cells. At the cell level, the flow rate was reduced to 5 mL/min/well using flow controllers to prevent cell damage. Aerosols were characterized in real time using a SMPS and a COP, to assess the size distribution and by gravimetric measurements, to assess the mass concentration. The deposition of NMs on the cells was assessed by performing QCM and ICP-MS measurements and TEM analysis, to assess the mass, shape, size and distribution of the NMs on the cells

To simulate inhalation in vitro and to study the toxicity of inhalable and poorly soluble metallic NMs on cells, a system using VitroCell® chambers was set up in our laboratory (Fig. 2). NM aerosols were generated by nebulization of NM suspensions, using a nebulizer. The deposition of NMs using similar VitroCell® systems was validated in several studies [36–39]. Moreover, it was shown that the deposited doses were sufficient to observe biological adverse effects, even at low exposure doses [36, 39]. To validate our system, four different metallic and poorly soluble NMs: three TiO_2_ (NM105, NM101, NM100) and one CeO_2_ NM (NM212), possessing different physicochemical characteristics (Table 1) were used. TiO_2_ and CeO_2_ NMs were selected because they are inhalable and commonly used in toxicity studies at the lung level [12, 20, 40].

**Table 1:** TiO_2_ and CeO_2_ physicochemical properties

		Critallinity	Primary particle size (nm)	Surface area, BET (m^2^/g)	Primary density (g/cm^3^)
TiO_2_	NM105	80 % anatase/20 % rutile	21	46.1	4.2
	NM101	anatase	8	316	3.9
	NM100	anatase	100	10	3.9
CeO_2_	NM212	cubic cerionite	29	27	7.2

#### Characterization of NM in aerosols

**Fig. 3:**
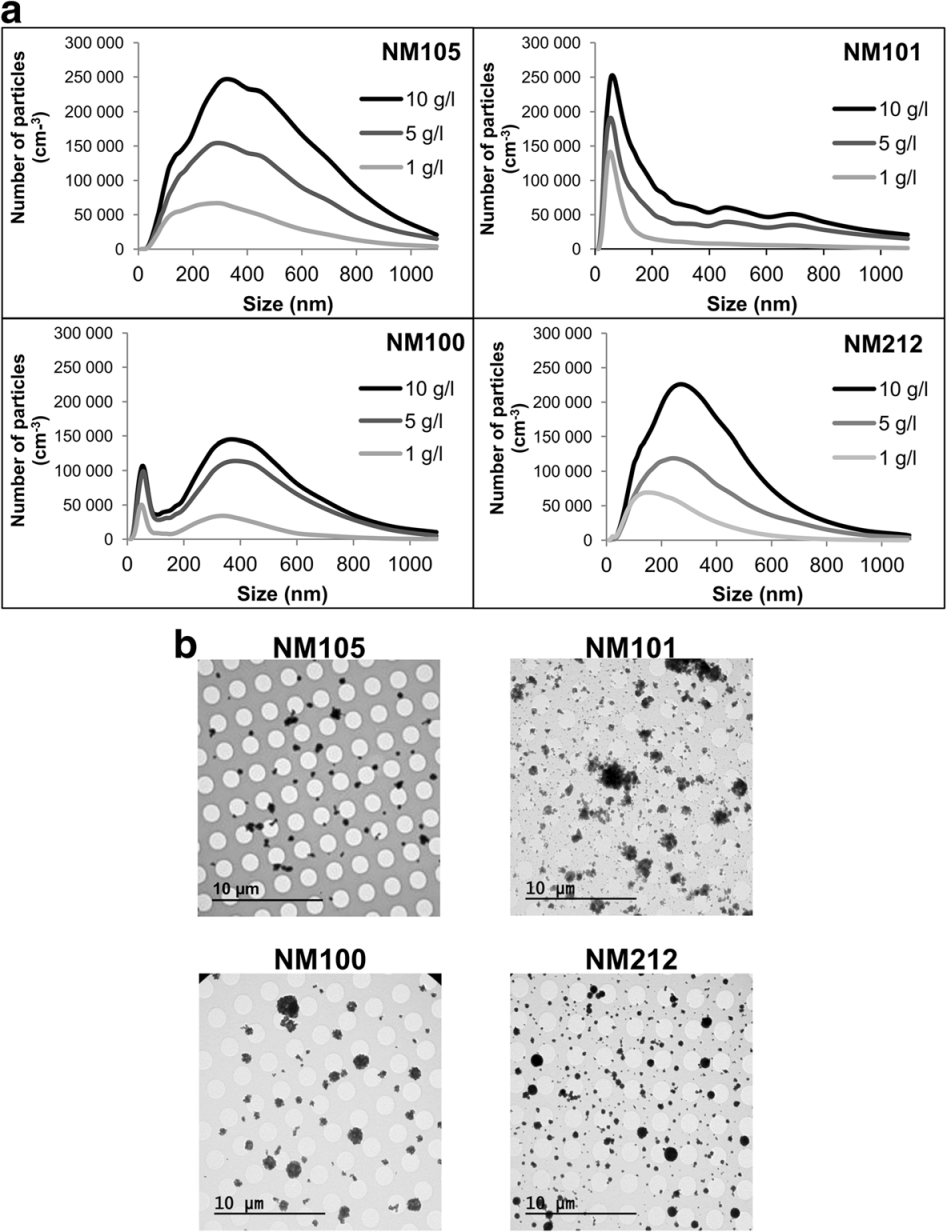
Number size distribution of the aerosols and respective deposition in inserts. Aerosols were generated by nebulization of suspensions of TiO_2_ (NMs 105, 101, 100) and CeO_2_ (NM212) at concentrations of 1 g/L (light grey), 5 g/L (dark grey), 10 g/L (black). The size distributions of the NMs in the aerosols were measured using a SMPS and an OPC, and particles ranged from 10 to 1095 nm and 300 to 34 000 nm, respectively (**a**). The deposition of the NMs on the TEM grids was assessed after exposure (**b**). TEM grids were placed on the apical side of inserts and exposed 3 h to aerosols generated with suspensions of 10 g/L in the nebulizer. After exposure, the grids were analyzed by TEM to assess the sizes, shapes and distributions of the deposited NMs

To evaluate the deposition of the NMs on the cells, we first characterized the NM aerosols that were generated in our system. By gravimetric measurements, NM mass concentrations of around 10, 50 and 100 mg/m^3^ were measured in the aerosols, for suspension concentrations in the nebulizer of 1, 5 and 10 g/L, respectively (Table 2). The mass size distributions of the aerosols were evaluated (Additional file 1: Figure S1) and the total aerosol volume concentrations were determined to assess the aerosol effective densities (Table 2). Consistently with the Cosnier et al. study [41], the aerosol effective densities were much lower than the NM primary densities. The number size distributions of the aerosols were evaluated (Fig. 3a): we observed some isolated primary-sized particles, but most particles were agglomerated, as indicated by higher Geometric Mean Diameters (GMDs) than the NM primary particle diameters (Table 2). This may explain why primary densities were low and why high Volumetric Mean Diameters (VMDs) were calculated (Table 2), indicating that most of the aerosol mass was due to agglomerates. Furthermore, we observed that the VMDs and GMDs of the NMs in the aerosols were correlated to the NM mass concentrations measured in the aerosols.

**Table 2:** Characterization of aerosols and deposition of NM on cells

Nebulizer (PALAS, AGK 2000)	TiO_2_ NM105			TiO_2_ NM101			TiO_2_ NM100			CeO_2_ NM212		
Suspension concentration (g/L)	1	5	10	1	5	10	1	5	10	1	5	10
Aerosol concentration (mg/m^3^) (Gravimetry)	7.9	52.5	105.7	7.1	35.1	75.3	10.6	55.4	101.8	10.6	56.7	113.5
Total aerosol volume concentration (um^3^/cm^3^) (SMPS + OPC)	18.6	83.3	133	11.8	66.4	114	11.7	55.3	118.4	10.8	45.8	101.3
Aerosol effective density^*a*^ (g/cm^3^)	0.42	0.63	0.79	0.90	0.83	0.89	0.60	0.63	0.64	0.98	1.24	1.12
Aerosol VMD^*b*^ (nm)	874	963	997	683	750	1060	1240	1360	1320	597	727	842
Volume geometric standard deviation	2.56	2.15	2.01	1.91	1.83	2.23	2.52	2.31	2.23	2.52	2.17	2.25
Aerosol GMD^*b*^ (nm)	196	234	249	61	74	85	289	319	317	135	190	210
Theoretical deposited mass^c^ (μg/cm^2^ in 3 h)	1.5	10.1	20.4	1.4	6.8	14.5	2.0	10.7	19.6	2.0	10.9	21.9
Deposited mass^*d*^(μg/cm^2^ in 3 h) (*n* = 4-7) (ICP-MS)	0.06 ± 0.01	0.66 ± 0.12	2.68 ± 0.6	0.22 ± 0.03	1.51 ± 0.11	3.15 ± 0.44	0.11 ± 0.01	0.50 ± 0.12	2.85 ± 0.30	0.22 ± 0.02	1.54 ± 0.08	3.26 ± 0.68
Deposited mass^*e*^(μg/cm^2^ in 3 h) (*n* = 3) (QCM)	0.11 ± 0.01	0.72 ± 0.06	1.07 ± 0.02	0.14 ± 0.01	1.08 ± 0.39	2.43 ± 0.21	0.44 ± 0.08	1.43 ± 0.53	3.21 ± 0.69	0.24 ± 0.05	1.37 ± 0.17	2.97 ± 0.64
Deposition efficiency^*f*^ (%) (ICP-MS)	4.1	6.5	13.2	15.8	22.4	21.7	5.2	4.7	14.5	10.7	14.1	14.9
Deposition efficiency^*f*^ (%)(QCM)	7.1	7.1	5.2	10.5	15.9	16.7	21.7	13.4	16.4	11.8	12.5	13.6

^*a*^Aerosol density = Aerosol concentration (gravimetry) / total aerosol volume concentration (SMPS + OPC)

^*b*^Geometric Mean Diameter (GMD) and Volumetric Mean Diameter (VMD) were measured with a Scanning Mobility Particle Sizer (SMPS) and an Optic Counter (OPC)

^*c*^Theoretical deposited mass calculated when assuming 100 % deposition on cells: theoretical deposited mass = mass concentration of aerosol/volume of aerosol passing through exposure chambers during exposure

^*d*^Deposited mass measured by Inductively Coupled Plasma - Mass Spectrometry (ICP-MS)

^*e*^Deposited mass measured using Quartz Cristal Microbalances (QCM)

^*f*^Deposition efficiency = Deposited mass measured / theoretical deposited mass calculated

#### Characterization of NM deposition on cells

After 3 h of exposure of the cells at the ALI, the size distribution of the particles deposited on the cell surfaces (Fig. 3) seemed to correlate with the size distribution of the particles in the aerosols. Thus, the complete range of particle sizes present in the aerosols was able to deposit on the cell surfaces during exposure (Fig. 3b). Furthermore, homogeneous distributions of the particles on the cell surfaces were observed (Fig. 3b). To assess the mass of NM deposited, QCM and ICP-MS techniques were used. Some differences in results were observed, which could be explained by technical differences between the two methods. In the QCM technique, NMs get deposited on an inert quartz surface instead of cells and there is no discrimination between NMs and potential contaminants in the deposited mass detected. However, the ICP-MS methodology reveals the mass of NMs deposited on the cell surface by direct dosage. For these reasons, we took the ICP-MS method as reference and the ICP-MS measurements were used to determine the deposited mass of the NMs on the cells at the ALI for the rest of the study. Deposited masses of around 0.1, 1, 3 μg/cm^2^ were measured for nebulized suspensions at concentrations of 1, 5 and 10 g/L, respectively (Table 1). The deposition efficiency on the cells was calculated based on the deposited mass, the mass concentration of the aerosol, the duration of exposure (3 h) and the flow rate in the VitroCell® chambers (5 mL/min). Depending on the NM physicochemical characteristics and on the initial suspension concentrations, mean depositions ranging from around 4 to 20 % were observed (Table 2).

The maximum deposition efficiency observed in our study was either higher or lower than those reported in several studies using similar exposure systems (4, 2, 1.1 % [37, 42, 43] and 70 % [36]). These discrepancies could be due to differences in the physicochemical characteristics of the NMs and aerosols generated or the methods used to characterize the deposition. Nevertheless, the maximum doses deposited in our system, although remaining high when put into perspective with real exposure scenarios [23], were low compared to those reachable through submerged exposure [23].

Devices allowing electrostatic deposition of charged particles [23, 34] or thermal precipitation [35] have been introduced to improve deposition efficiency. For example, Panas et al. [38] increased the deposition percentage of SiO_2_ NMs of 50 nm from 0.5 to 11 % using an electromagnetic field. However, it remains unclear whether modification of the overall particle charge may alter the particle-cell interactions. Furthermore, the mean deposition rates obtained with our system were sufficient for further toxicological assessments and we decided to test the NM toxicities without using this approach.

#### Cellular models used to assess toxicity of NM at the air-liquid interface

We assessed the toxicity of TiO_2_ and CeO_2_ NMs at the ALI with our aerosol exposure system. Two cellular models of the alveolar epithelium were chosen. Monocultures of A549 cells, which is one of the most studied alveolar epithelial cell line, were used for the ability of these cells to form a cell layer (although without functional tight junctions), grow at the ALI in inserts and secrete surfactant [44, 45]. This cell line was successfully exposed at the ALI to NM aerosols in several studies in which similar exposure systems were used [36, 38]. A co-culture model of A549 cells and THP-1 cells, differentiated into macrophages, was also used to mimic complex cell-cell interactions and communications occurring in vivo. This model was chosen to represent the complex alveolar structure comprising macrophages, in close contact with alveolar cells, which are involved in the mechanisms of defense against particles [46, 47]. Circulating in the lumen of the alveolar space, macrophages have the ability to produce pro-inflammatory markers and internalize particles, underlining their relevance in the study of host responses to NMs [46, 47]. To mimic physiology, a ratio of ten A549 cells to one THP-1 cell was used, which is among the highest pneumocyte to macrophage ratio observed in normal human lungs [48].

To enable ALI exposure to NM aerosols, the cellular models were grown in 0.4 μm pore inserts. In this configuration, after reaching confluence it was possible to keep the A549 monocultures at the ALI, in the presence (for co-cultures) or not of differentiated THP-1 cells, without observing non-physiological medium translocation from the basolateral to the apical compartment. To maintain physiological conditions and prevent damage from exposure to air, the cultures were exposed to aerosols with 90 % humidity and exposure times of 3 h with 5 mL/min flow rates were used. In these conditions, no decrease in mono or co-culture viability was observed, compared to control cells kept at the ALI in the incubator (Additional file 1: Figure S2).

#### NM toxicity at the air-liquid interface

**Fig. 4:**
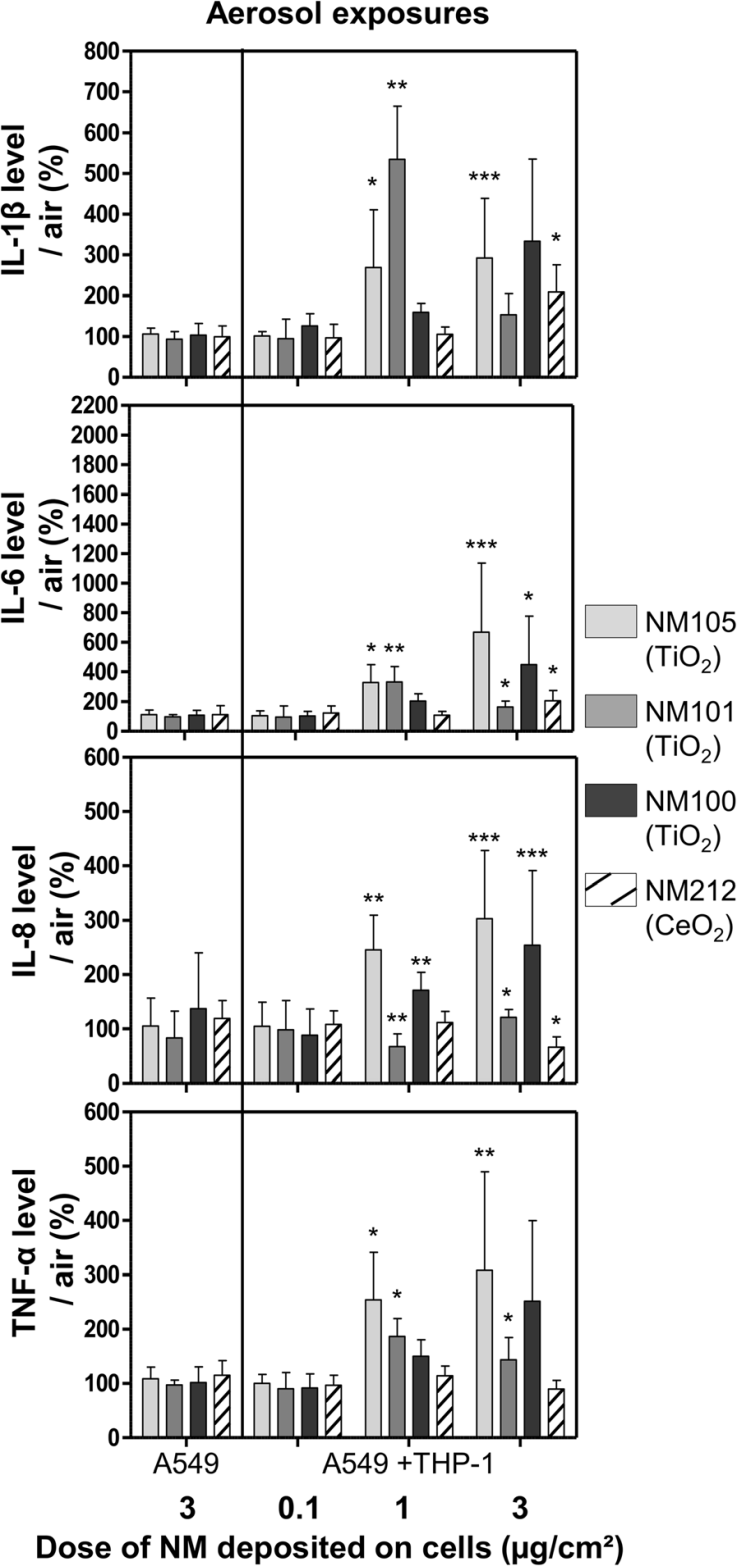
Levels of pro-inflammatory mediators IL-1β, IL-6, IL-8 and TNF-α in culture medium of cells exposed at the ALI to aerosols. Mono (A549) and co-cultures (A549 + THP-1) were exposed for 3 h at the ALI to aerosols of TiO_2_ (NM105, NM101, NM100) and CeO_2_ (NM212) or air and kept in the incubator at the ALI for 21 h, with NMs deposited on their surface. Deposited doses were around 0.1, 1, 3 μg/cm^2^. At 24 h, IL-1β, IL-6, IL-8 and TNF-α levels in the culture medium were measured by ELISA multiplex at the basal side. For exposures at the ALI, specific air and positive controls (LPS 20 μg/mL) (not shown on the graph) were used for each NM used and for each concentration tested. Data represent the mean ± Standard Deviation (SD) of three independent experiments. A Kruskal-Wallis test followed by Dunn’s post-hoc test were performed to compare treated groups to controls (*p < 0.05; **p < 0.01; ***p < 0.001)

**Fig. 5:**
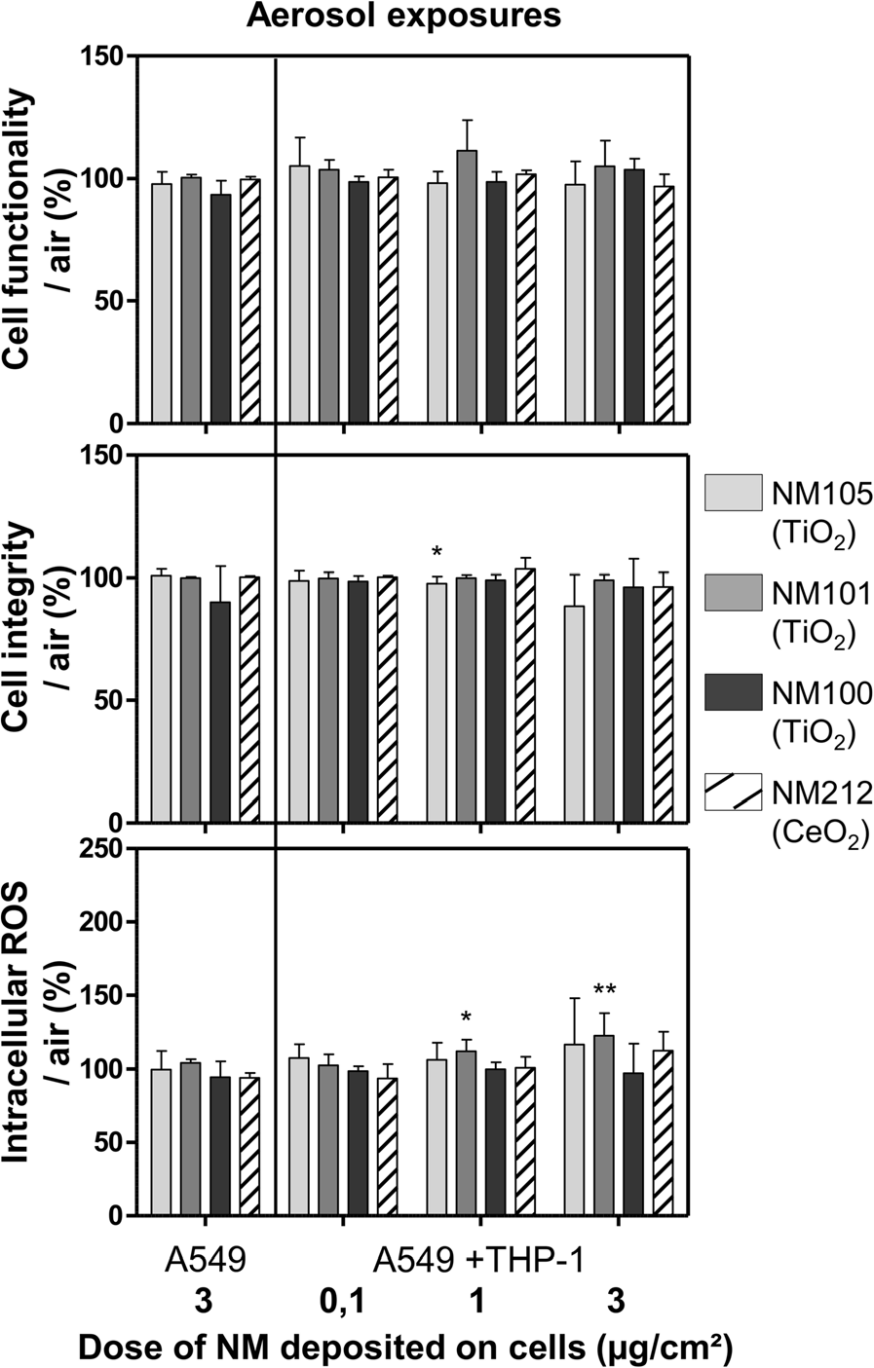
Functionality, integrity and intracellular ROS levels of cells exposed at the ALI to aerosols. Mono (A549) and co-cultures (A549 + THP-1) were exposed for 3 h at the ALI to aerosols of TiO_2_ (NM105, NM101, NM100) and CeO_2_ (NM212) or air and kept at the ALI for 21 h in the incubator, with NMs deposited on their surface. Deposited doses were around 0.1, 1, 3 μg/cm^2^. At 24 h, Alamar blue® and LDH assays were performed to assess functionality and integrity of the cells, respectively. A DCF assay was performed to measure intracellular ROS levels and H_2_O_2_ (1 mM) was used as positive control for the DCF assay (not shown on the graph). Specific air and incubator controls (cells kept in the incubator) (not shown on the graph) were used for each NM used and for each concentration tested. Data represent the mean ± SD of three independent experiments. A Kruskal-Wallis test followed by Dunn’s post-hoc test were performed to compare treated groups to control (*p < 0.05; **p < 0.01; ***p < 0.001)

To assess the potential adverse effects generated by TiO_2_ and CeO_2_ NMs, mono and co-cultures were exposed to one (about 3 μg/cm^2^) and three doses (about 0.1 μg/cm^2^, 1 μg/cm^2^ and 3 μg/cm^2^) of NMs, respectively (Table 2). These final doses were achieved by exposing the cells at the ALI to the aerosols of NMs continuously for 3 h. After apical NM deposition, the cells were kept at the ALI in the incubator during 21 h with fresh medium containing 10 % Fetal Bovine Serum (FBS) in the basolateral compartment. Twenty-four hours after exposure, we generally observed significant biological adverse effects in the co-culture models and none in the monocultures. Across the different biological assays performed, adverse effects were detected at medium and high doses of NMs (1 and 3 μg/cm^2^) (Figs. 4 and 5) and none were observed at doses of 0.1 μg/cm^2^.

Among all the markers tested, the pro-inflammatory mediators were the most sensitive. In the co-cultures, all the cytokines tested were high above the quantification limit. In the monocultures, IL-1β levels were between detection and quantification limits, IL-6 and TNF-α levels were just above the quantification limit and IL-8 levels were largely above the quantification limit. We observed significant differences in cytokine levels in the co-cultures with all NMs tested, compared to control (Fig. 4). TiO_2_ NMs105 and 101 triggered pro-inflammatory responses at lower doses than TiO_2_ NM100 and CeO_2_ NM212. After exposure to aerosols of TiO_2_ NM105, we observed increased levels of IL-1β, IL-6, IL-8 and TNF-α at doses of 1 and 3 μg/cm^2^. With TiO_2_ NM101, we observed significant increases in IL-1β, IL-6, TNF-α levels at doses of 1 and 3 μg/cm^2^ and in IL-8 levels at 3 μg/cm^2^, compared to control. Intriguingly, we observed lower levels of response to TiO_2_ NM101 at high doses than at medium doses. Although cytokines were dosed in the basolateral compartment to avoid bias due to potential interactions with the NMs, we cannot rule out the possibility that apical cytokines coated with NMs at the apical side may not have translocated to the basal compartment inducing variability in the results at high doses. With TiO_2_ NM100, we observed a significant increase in IL-8 levels at doses of 1 μg/cm^2^ and a significant increase of IL-6 levels at doses of 3 μg/cm^2^, compared to control. With regards to CeO_2_ NM212, we observed a significant increase in IL-1β and IL-6 levels and a significant decrease in IL-8 levels at doses of 3 μg/cm^2^, compared to control.

Cell functionality was not affected across the conditions. Regarding cell integrity, we observed a slight but statistically significant decrease in cell integrity (around 5 %) in the co-cultures exposed to medium doses of NM105, but not with the other NMs (Fig. 5). However, probably due to high variability, we didn’t observe a significant loss of integrity in the co-cultures exposed to high doses of NM105. The integrity of the cells was not impaired with the other NMs (Fig. 5). Finally, a significant increase in intracellular ROS levels was observed in co-cultures exposed to high doses of NM101 but not with the other NMs (Fig. 5).

The potential adverse effects of poorly soluble NMs were assessed at the ALI in a few other studies. For example, in two linked studies [49, 50], the cytotoxicity of TiO_2_ NM105 was evaluated in A549 cells using a CULTEX® Radial flow system module. With a deposited mass of 25 μg/cm^2^ per 15 min on the cells during 15, 30 and 60 min, they showed a strong decrease in cell viability 24 h after exposure, of about 50, 60 and 70 %, respectively. Using an electrode device to enhance deposition, Panas et al. [38] exposed A549 cells to aerosols of Aerosil200 (SiO_2_) and SiO_2_-50 with deposited doses of 52 μg/cm^2^ and 117 μg/cm^2^, respectively. They showed increased lactate deshydrogenase (LDH) and IL-8 release after exposure to Aerosil200 and increased IL-8 release after exposure to SiO_2_-50.

To conclude, it was shown in several studies that ALI systems can be used to assess the toxicity of soluble NMs in monocultures and co-cultures [36, 39, 51, 52] with deposited doses of about 1-3 μg/cm^2^. Assessment of poorly soluble NM toxicity was also proven to be feasible with alveolar epithelial cells in monocultures, by increasing the deposited doses to around 25–100 μg/cm^2^ [38, 49]. Our results indicate that it is also possible to evaluate the toxicity of metallic and poorly soluble NMs at the ALI at lower deposited doses, by using more sensitive cell models like the co-cultures. The enhanced sensitivity of co-culture models including macrophages compared to monocultures of alveolar epithelial cells was also observed in several studies in submerged conditions [53–55]. More physiological culture models such as human primary alveolar epithelial cells could also be used. However, these are not commercially available and their use requires to have access to human biopsies [32].

### Exposure in submerged conditions to suspensions of NM

Since we could only observe biological adverse effects with the co-cultures after exposure to aerosols of NMs at the ALI, we focused on this model for the following experiments. The co-cultures were exposed in submerged conditions to suspensions of TiO_2_ NMs 105, 100, 101 and CeO_2_ NM212 in inserts and plates with culture medium containing 10 % FBS. In inserts, the final deposited dose was achieved within 3 h, after which the suspensions were replaced with fresh medium and the cells were kept in the incubator during the remaining 21 h with the deposited NMs on their surface. In plates, the final deposited dose was achieved within 24 h.

#### Characterization of suspensions and deposited doses on cells

For accuracy, we characterized the NMs in suspension and estimated the real mass deposited on the cells after 3 h of exposure in inserts and 24 h of exposure in plates, using the in vitro sedimentation diffusion and dosimetry (ISDD) model [30] (Table 3). First, Dynamic Light Scattering (DLS) measurements were performed in 2.56 mg/mL sonicated stock suspensions in Milli-Q water and in 0.4 mg/mL suspensions in culture medium (Additional file 1: Figure S3), to assess the size distribution and hydrodynamic diameter of the NMs (Table 3). We observed that the NMs were all polydispersed and that most of the particles were agglomerated in suspension, with sizes ranging approximately from 100 to around 1000 nm (Additional file 1: Figure S3). Furthermore, we observed similar distributions in the Milli-Q water and the culture medium. The effective densities of the NMs in culture medium were measured following the Volumetric Centrifugation Method (VCM) developed by Deloid and coworkers [56] (Table 3). Once the NM suspensions were characterized, we used these values to estimate the mean deposited fractions of the NMs on the cells after 3 h and 24 h of exposure, using the ISDD model (Table 3). It is important to note that for each NM we assumed that all the particles were agglomerated, had the same size in suspension and had the same density. To estimate more precisely the deposition, which depends highly on the size and effective density, it would have been necessary to measure the sizes and effective densities of all the agglomerates separately, as mentioned by Deloid et al. [57].

**Table 3:** Characterization of suspensions and deposition of NM on cells

	Hydrodynamic diameter^*a*^, Z-average (nm) (*n* = 6)	Effective density^*b*^ (g/cm^3^) (*n* = 3)	Deposited fraction after 24 h in plates^*c*^	Deposited fraction after 3 h in inserts^*c*^
NM105	381.1	1.4	28.5 %	8.6 %
NM101	660.9	1.586	100.0 %	20.0 %
NM100	353.0	1.938	70.0 %	13.6 %
NM212	240.7	1.9701	37.8 %	11.0 %

^*a*^DLS measurement

^*b*^Measured after centrifugation, following the VCM developed by Deloid et al.[56]

^*c*^Estimated using the ISDD model

Initial concentrations in suspensions were adjusted according to the estimated deposited fractions to determine the real dose deposited on the cells (Table 4). As shown by Deloid et al., we observed that the particles were able to settle faster when the hydrodynamic diameter and the effective density were higher. Furthermore, as it was shown that NMs could interfere in assays [58–60] leading to misinterpretation of results, we assessed the potential interactions between the NMs and the cytokine and LDH assays (Additional file 1: Figure S4).

**Table 4:** Dose deposited in submerged conditions in function of nominal concentration in suspensions

		24 h deposition in plates				3 h deposition in inserts		
TiO_2_ NM105	Nominal dose (μg/mL)	10	50	100	200	54.5	163.5	544.9
	Nominal dose (μg/cm^2^)	2.5	12.5	25	50	11.7	35.0	116.7
	Estimated dose using the ISDD model (μg/cm^2^)	0.7	3.6	7.1	14.3	1	3	10
TiO_2_ NM101	Nominal dose (μg/mL)	4	10	50	100	23.4	70.1	233.5
	Nominal dose (μg/cm^2^)	1	2.5	12.5	25	5.0	15.0	50.0
	Estimated dose using the ISDD model (μg/cm^2^)	1.0	2.5	12.5	25.0	1	3	10
TiO_2_ NM100	Nominal dose (μg/mL)	4	10	50	100	34.3	102.9	343.1
	Nominal dose (μg/cm^2^)	1	2.5	12.5	25	7.3	22.0	73.5
	Estimated dose using the ISDD model (μg/cm^2^)	0.7	1.8	8.8	17.5	1	3	10
CeO_2_ NM212	Nominal dose (μg/mL)	10	50	100	200	42.5	127.4	424.5
	Nominal dose (μg/cm^2^)	2.5	12.5	25	50	9.1	27.3	90.9
	Estimated dose using the ISDD model (μg/cm^2^)	0.9	4.7	9.5	18.9	1	3	10
Tested doses about (μg/cm^2^)		1	3	10	20	1	3	10

#### NM toxicity in submerged conditions

Co-cultures were exposed to suspensions of NMs in inserts using similar culture conditions and exposure kinetics to the air-liquid interface, to assess whether the cells were more sensitive to NMs when exposed to aerosols at the ALI. Cells were exposed for 3 h to NM suspensions to achieve deposited doses of around 1, 3, and 10 μg/cm^2^ (Table 4). Cells were then kept in the incubator with fresh medium during the remaining 21 h with the deposited NMs on their surface, and biological adverse effects were assessed at 24 h.

**Fig. 6:**
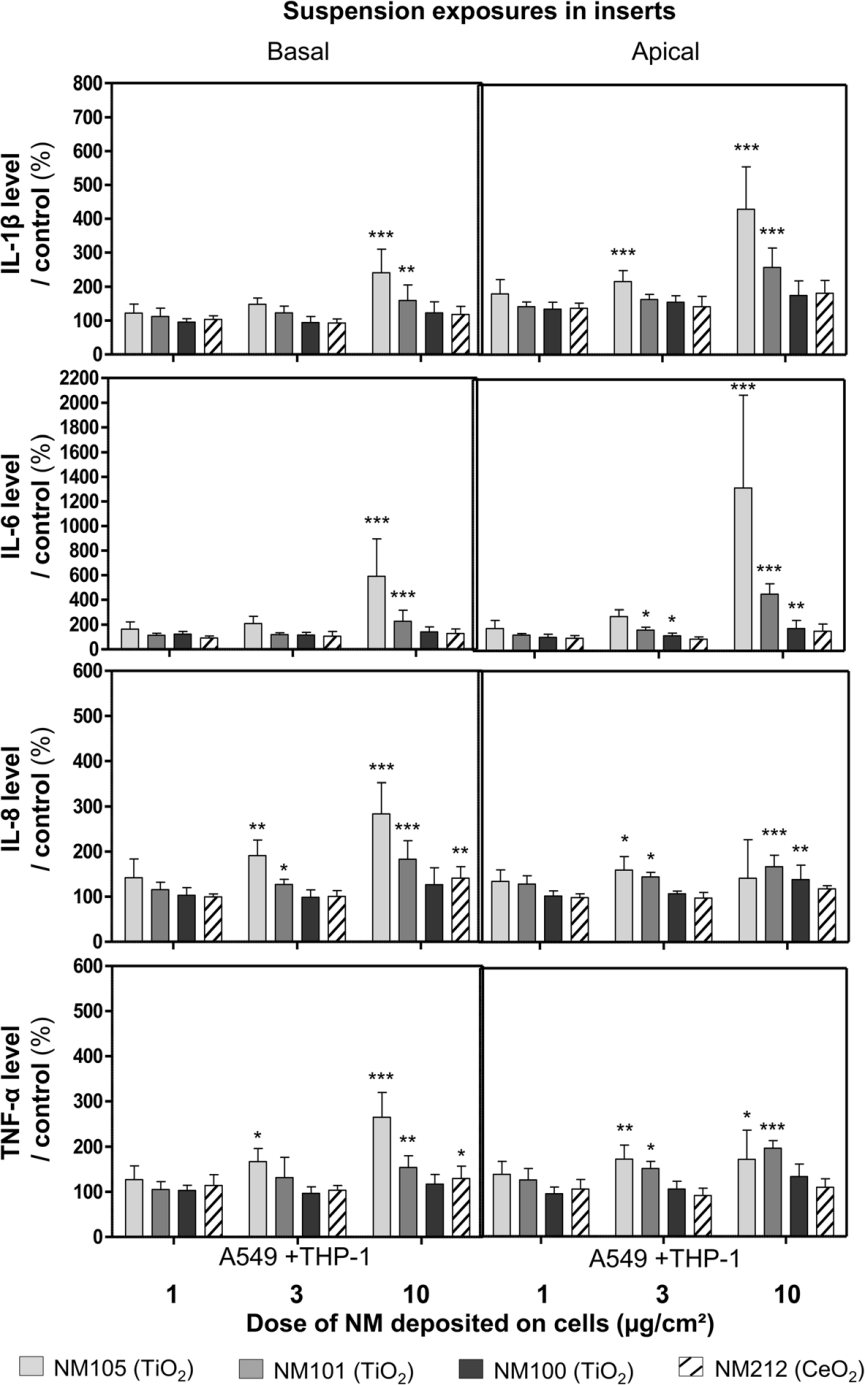
Levels of pro-inflammatory mediators IL-1β, IL-6, IL-8 and TNF-α in culture medium of cells exposed in submerged conditions in inserts. Co-cultures (A549 + THP-1) were exposed in inserts for 3 h to suspensions of TiO_2_ (NM105, NM101, NM100) and CeO_2_ (NM212), to achieve deposited doses of around 1, 3 and 10 μg/cm^2^. Suspensions were then replaced by fresh medium and cells were kept for 21 h at the incubator with NMs deposited on their surface. At 24 h, IL-1β, IL-6, IL-8 and TNF-α levels were measured by ELISA multiplex in cell culture medium (apical and basal sides). A specific control (cells exposed to culture medium) and positive control (LPS 20 μg/mL) (not shown on the graph) were used for each NM used. Data represent the mean ± Standard Deviation (SD) of three independent experiments. A Kruskal-Wallis test followed by Dunn’s post-hoc test were performed to compare treated groups to controls (*p < 0.05; **p < 0.01; ***p < 0.001)

The levels of the pro-inflammatory mediators IL-1β, IL-6, IL-8 and TNF-α were assessed after submerged exposure in inserts, and similarly to at the ALI we generally observed significant effects at lower doses with TiO_2_ NMs 105 and 101 than with TiO_2_ NM100 and CeO_2_ NM212 (Fig. 6). With NM105, we observed significant increases in IL-1β, IL-8 and TNF-α levels at doses of 3 and 10 μg/cm^2^ and 10 μg/cm^2^ for IL-6. Significant effects were observed with NM101 at 3 and 10 μg/cm^2^ for IL-6, IL-8 and TNF-α and at 10 μg/cm^2^ for IL-1β. Significant inductions were observed for IL-6 and IL-8 with NM100, at doses of 3 and 10 μg/cm^2^ and 10 μg/cm^2^, respectively. Finally, we observed significant effects only with IL-8 and TNF-α, at doses of 10 μg/cm^2^ with NM212.

**Fig. 7:**
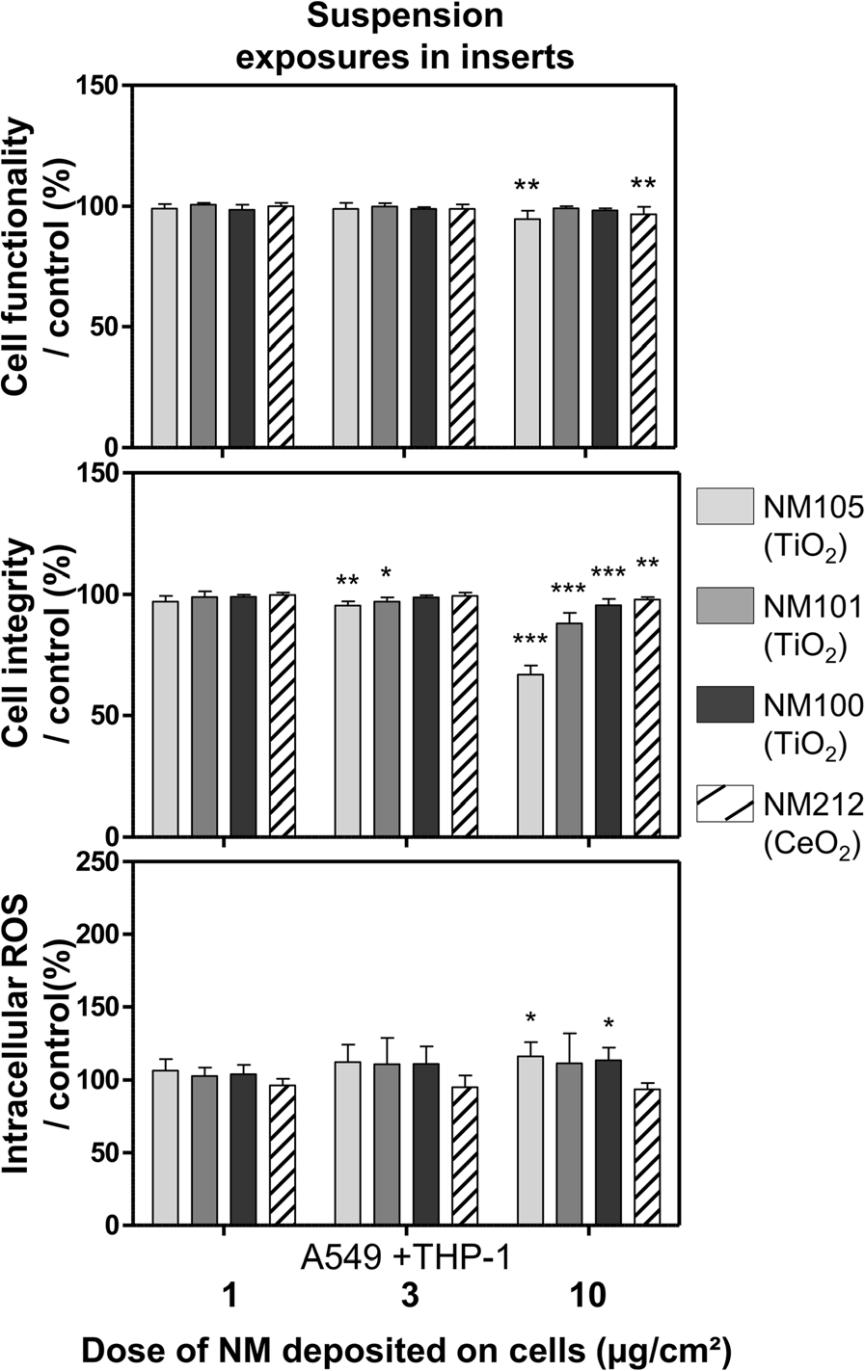
Functionality, integrity and intracellular ROS levels of cells exposed in submerged conditions in inserts. Co-cultures (A549 + THP-1) were exposed in inserts for 3 h to suspensions of TiO_2_ (NM105, NM101, NM100) and CeO_2_ (NM212), to achieve deposited doses of around 1, 3 and 10 μg/cm^2^. Suspensions were then replaced by fresh medium and cells were kept for 21 h at the incubator with NMs deposited on their surface. At 24 h, Alamar blue® and LDH assays were performed to assess functionality and integrity of the cells, respectively. A DCF assay was performed to measure intracellular ROS levels and H_2_O_2_ (1 mM) was used as positive control for the assay (not shown on the graph). Data represent the mean ± SD of three independent experiments. A Kruskal-Wallis test followed by Dunn’s post-hoc test were performed to compare treated groups to controls (*p < 0.05; **p < 0.01; ***p < 0.001)

Cell functionality and integrity were measured to evaluate cell viability (Fig. 7). We observed slight (below 5 %) but significant decreases in cell functionality at the dose of 10 μg/cm^2^ with the NMs 105 and 212 only, compared to control. However, we observed significant decreases in cell integrity with all the NMs tested: at doses of 3 and 10 μg/cm^2^ for the NMs 105 and 101 and at doses of 10 μg/cm^2^ for the NMs 100 and 212. Intracellular ROS levels were also measured and we observed significant increases only after exposure to doses of 10 μg/cm^2^ with NMs 105 and 100 (Fig. 7).

**Fig. 8:**
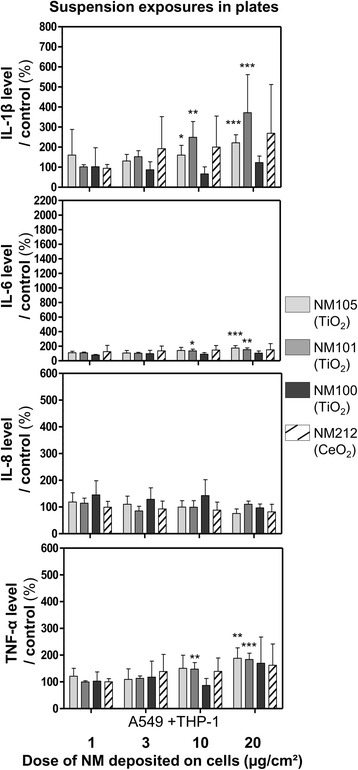
Levels of pro-inflammatory mediators IL-1β, IL-6, IL-8 and TNF-α in the culture medium of cells exposed in submerged conditions in plates. Co-cultures (A549 + THP-1) were exposed in plates for 24 h to suspensions of TiO_2_ (NM105, NM101, NM100) and CeO_2_ (NM212), to achieve deposited doses of around 1, 3 and 10 and 20 μg/cm^2^. IL-1β, IL-6, IL-8 and TNF-α levels were measured by ELISA multiplex in the culture medium. A specific control (cells exposed to culture medium) and positive control (LPS 20 μg/mL) (not shown on the graph) were used for each NM used Data represent the mean ± SD of three independent experiments. A Kruskal-Wallis test followed by Dunn’s post-hoc test were performed to compare treated groups to controls (*p < 0.05; **p < 0.01; ***p < 0.001)

**Fig. 9:**
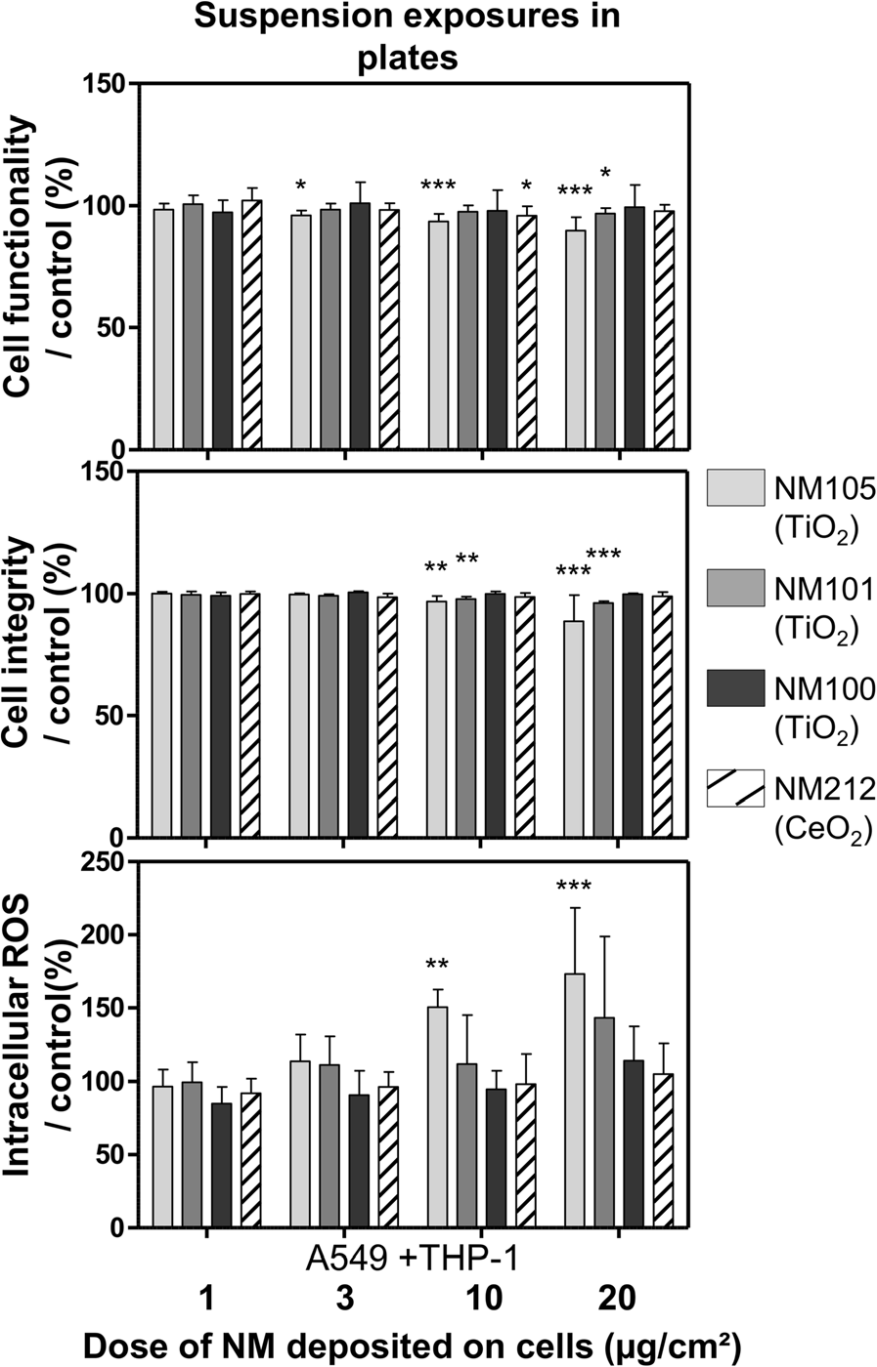
Functionality, integrity and intracellular ROS levels of cells exposed in submerged conditions in plates. Co-cultures (A549 + THP-1) were exposed in plates for 24 h to suspensions of TiO_2_ (NM105, NM101, NM100) and CeO_2_ (NM212), to achieve deposited doses of around 1, 3 and 10 and 20 μg/cm^2^. Alamar blue® and LDH assays were performed to assess functionality and integrity of the cells, respectively. A DCF assay was performed to measure intracellular ROS levels and H_2_O_2_(1 mM) was used as positive control for the assay (not shown on the graph). Data represent the mean ± SD of three independent experiments. A Kruskal-Wallis test followed by Dunn’s post-hoc test were performed to compare treated groups to controls (*p < 0.05; **p < 0.01; ***p < 0.001)

Co-cultures were also exposed in plates for 24 h to deposited doses of about 1, 3, 10 and 20 μg/cm^2^ (Table 4), to compare the ALI results with those obtained by classic submerged protocols (Figs. 8 and 9). In plates, we only observed significant biological effects with the TiO_2_ NMs 105 and 101, compared to control. We observed significant increases in the pro-inflammatory mediators IL-1β and IL-6 at doses of 10 and 20 μg/cm^2^ for NMs 105 and 101 (Fig. 8). Significant increases in TNF-α levels at doses of 10 and 20 μg/cm^2^ for NM101 and 20 μg/cm^2^ for the NM105 were also observed, compared to control. Nevertheless, we didn’t observe any significant effects on IL-8 levels, probably due to NM-cytokine interactions (Additional file 1: Figure S4). Regarding cell functionality, decreases were observed in cells exposed to suspensions of NM105 (at doses of 3, 10 and 20 μg/cm^2^) and NM101 (at doses of 20 μg/cm^2^) (Fig. 9). We observed a loss in cell integrity at doses of 10 and 20 μg/cm^2^ (Fig. 9). Finally, we observed significant increases in intracellular ROS levels at doses of 10 and 20 μg/cm^2^ with NM105 only (Fig. 9).

As reported in several studies, we observed that TiO_2_ NM [20, 40] and CeO_2_ NM [61, 62] were slightly toxic but could induce oxidative stress, inflammation, and cytotoxicity after exposure to high concentrations in suspension [12].

### Comparison between air-liquid interface and submerged results

We observed significant pro-inflammatory effects in the co-cultures both at the ALI and in submerged conditions (Table 5). However, we observed less significant impacts in cytotoxicity and oxidative stress (Table 6). To provide accurate comparisons between ALI and submerged exposures, the potential interactions between the NMs in suspension and the LDH and cytokine assays were assessed in cell free conditions, as described in the materials and methods section. No interactions between the LDH assay and the NMs were observed (Additional file 1: Figure S4a). Although interactions were detected between the pro-inflammatory markers and the NMs (Additional file 1: Figure S4b), these interactions did not prevent appropriate data interpretation. Release of pro-inflammatory mediators by the cells appeared to be the most sensitive indicator of biological adverse effects to the NMs at 24 h. For this reason, we focused on the pro-inflammatory responses to the NMs to perform the following comparisons between ALI and submerged exposures.

**Table 5:** Lowest observed adverse effect levels (LOAELs in μg/cm^2^) determined with the pro-inflammatory effects for each exposure method used

	IL-1β			IL-6			IL-8			TNF-α		
	ALI^a^	Subm insert^b^	Subm plate^c^	ALI^a^	Subm insert^b^	Subm plate^c^	ALI^a^	Subm insert^b^	Subm plate^c^	ALI^a^	Subm insert^b^	Subm plate^b^
NM105	1	3	10	1	10	20	1	3	Ø	1	3	20
NM101	1	10	10	1	3	10	1	3	Ø	1	3	10
NM100	Ø	Ø	Ø	3	3	Ø	1	Ø	Ø	Ø	Ø	Ø
NM212	3	Ø	Ø	3	Ø	Ø	1	10	Ø	Ø	10	Ø

^a^Exposure at the air-liquid interface in inserts (ALI)

^b^Exposure in submerged conditions in inserts

^c^Exposure in submerged conditions in plates

Ø No effects measured at tested doses

**Table 6:** LOAELs in μg/cm^2^ determined with the cytotoxity and oxidative stress effects for each exposure method used

	Alamar blue			LDH			DCFDA		
	ALI^a^	Subm insert^b^	Subm plate^c^	ALI^a^	Subm insert^b^	Subm plate^c^	ALI^a^	Subm insert^b^	Subm plate^c^
NM105	1	10	3	1	3	10	Ø	10	10
NM101	Ø	Ø	20	Ø	3	10	1	Ø	Ø
NM100	Ø	Ø	Ø	Ø	10	Ø	Ø	10	Ø
NM212	Ø	10	10	Ø	10	Ø	Ø	Ø	Ø

^a^Exposure at the air-liquid interface in inserts (ALI)

^b^Exposure in submerged conditions in inserts

^c^Exposure in submerged conditions in plates

Ø No effects measured at tested doses

#### Comparisons according to deposited doses

We observed significant pro-inflammatory responses in cultures exposed to lower deposited doses at the ALI compared to submerged exposures (Table 5). Moreover, in submerged conditions, we observed significant effects at lower doses in inserts compared with plates (Table 5). Nevertheless, in vitro effects were observed at extremely high doses (at least 100 fold higher) compared to realistic human exposure scenarios [23].

We determined the Lowest Observed Adverse Effect Levels (LOAELs) for significant pro-inflammatory levels. For TiO_2_ NMs 105 and 101 we observed LOAELs at doses of 1 μg/cm^2^ at the ALI, at 3 or 10 μg/cm^2^ in submerged conditions in inserts and at 10 or even 20 μg/cm^2^ in plates. After exposure to TiO_2_ NM100 or CeO_2_ NM212, we observed significant adverse effects at doses of 1 or 3 μg/cm^2^ at the ALI, at doses of 10 μg/cm^2^ in submerged conditions in inserts but no effects after exposure in plates, even with the maximum concentration tested. Based on these results, we provided a ranking of the four NMs used in our study according to the LOAELs observed. Generally, the rankings were similar whatever the exposure method used. We observed significant adverse effects at lower doses with TiO_2_ NMs 105 and 101, compared to TiO_2_ NM100 and CeO_2_ NM212. This was in agreement with both in vitro and in vivo literature observations. The mass based toxic effects observed depend on the NM primary particle size (surface area) [12]. Indeed, despite differences regarding their crystalline phases, TiO_2_ NM105 (20 % anatase, 80 % rutile) and NM101 (100 % anatase), with primary particle sizes of 21 and 8 nm respectively, were more toxic than NM100, which has a primary particle size of 100 nm. The observed toxic effects also depend on the NM chemical composition and surface properties [12]. We observed that CeO_2_ NM212, with a primary particle size of 29 nm, appeared as toxic as TiO_2_ NM100, which has a primary particle size of 100 nm and less toxic than NM105, which has a similar primary particle size.

**Fig. 10:**
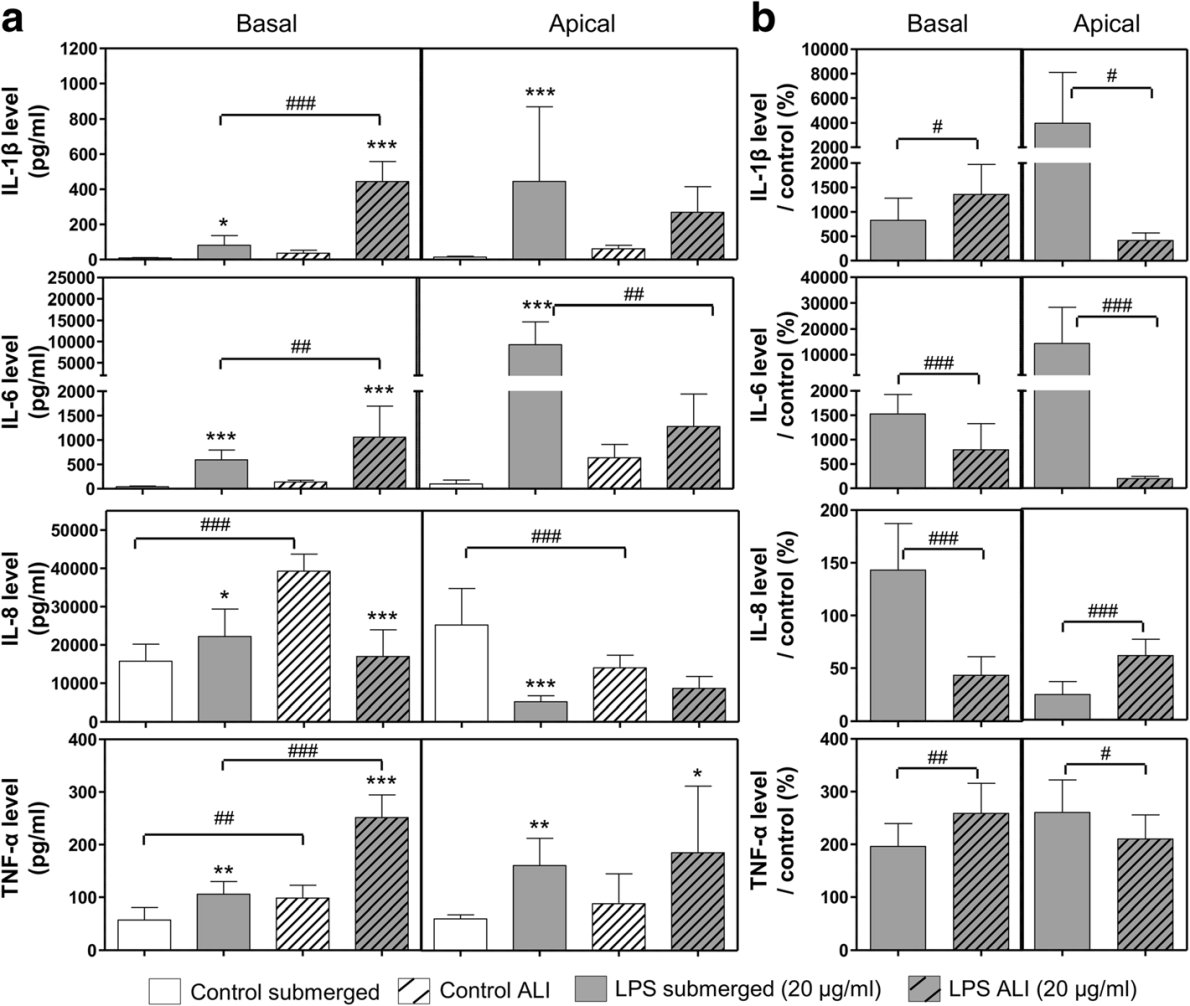
levels of pro-inflammatory mediators after stimulation with LPS (20 μg/mL). Co-cultures were stimulated at the ALI or in submerged conditions in inserts with 20 μg/ml of LPS at the basal side for 21 h. Levels of IL-1β, IL-6, IL-8 and TNF-α were assessed in culture medium at basal side and in culture medium or washing liquid at the apical side for submerged and ALI exposures, respectively. The control for ALI exposures was cells exposed at the ALI to air for 3 h in the exposure system and kept at the ALI for 21 h with fresh medium in the incubator. The control for submerged exposure was cells kept in submerged condition with fresh medium for 21 h. IL-1β, IL-6, IL-8 and TNF-α secretions were measured by ELISA multiplex in the cell culture medium. Results were first expressed in concentrations (pg/mL), to assess whether cells secreted similar amounts of cytokines at the ALI and in submerged conditions in inserts, after stimulation (**a**). Because we observed more basal secretion at the ALI (secretion by non stimulated cells), the data was also expressed in cytokine levels compared to control (**b**), to compare ALI and submerged results more accurately. Data represent the mean ± SD of three independent experiments. A two-way Anova followed by a Bonferroni post-hoc test were performed to compare treated groups to their respective controls (*p < 0.05; **p < 0.01; ***p < 0.001) or to compare ALI and submerged exposures (^#^ *p* < 0.05; ^##^ *p* < 0.01; ^###^ *p* < 0.001)

To understand why significant effects were observed at lower deposited doses at the ALI than in submerged conditions, we attempted to evaluate whether the cells cultivated at the ALI were more sensitive to xenobiotics in general than cells cultivated in submerged conditions. We compared the release of pro-inflammatory mediators in the co-culture model in insert at the ALI and in submerged conditions. We stimulated cells cultured at the ALI and in submerged conditions with a dose of 20 μg/mL of Lipopolysaccharide (LPS) in the basolateral compartment (Fig. 10). The stimulation was effective and significant differences in cytokine levels were observed for all the cytokines tested, compared to non-stimulated controls. In ALI conditions we observed cytokine inductions only at the basolateral side, except for TNF-α (Fig. 10a) and in submerged exposures we observed inductions both at the apical and basal sides (Fig. 10a). Moreover, we observed significantly higher levels of pro-inflammatory mediators at the basolateral side but not at the apical side after stimulation at the ALI compared to submerged conditions (Fig. 10a). This can be partly attributable to higher basal secretion at the basolateral side at the ALI. Indeed, when the data was normalized to non-stimulated cellular secretion, the increase of cytokine secretion at the ALI was less clear, compared to submerged exposure (Fig. 10b). At the ALI, we dosed significantly more cytokines at the basolateral side for IL-1β and TNF-α, but significantly less for IL-6, compared to submerged conditions. On the contrary, in submerged conditions we dosed significantly more cytokines at the apical side compared to at the ALI. For IL-8, stimulation with LPS induced an unexpected significant drop in secretion compared to control (Fig. 10a) with significantly less cytokines at the basolateral side and more at the apical side in ALI compared to submerged conditions (Fig. 10b). In summary, there seemed to be opposite trends in the polarity of cytokine secretion between the exposure conditions, with more basolateral secretion at the ALI and more apical secretion in submerged conditions. Nevertheless, after taking into account the different cellular responses observed in the ALI and submerged conditions after exposure to LPS, we observed that the levels of induction were similar. We concluded, therefore, that there was no difference in sensitivity to xenobiotics that could explain the higher adverse effects observed in the cells at the ALI after exposure to NMs (Table 5).

We also assessed whether the differences observed between the ALI and submerged conditions could be explained by differences in cell densities. In their study, Lenz et al. [63] observed a strong correlation between exposures at the ALI in inserts and submerged conditions in inserts and in plates, when expressing results in dose per cell units. We measured the number of cells in the inserts and in the plates during exposure to the NMs. We counted similar numbers of cells at the ALI (599 000 cells/cm^2^) and in submerged conditions (608 000 cells/cm^2^) in inserts, and fewer cells in submerged conditions in plates (226 000 cells/cm^2^). Thus, by expressing results in doses per cell, the differences observed in LOAELs remained similar between conditions in inserts. Moreover, lower cell densities in submerged conditions in plates conferred higher LOAELs when expressing the results in doses per cell instead of doses per surface thus increasing the differences observed between the ALI and classical submerged conditions. In our study, cells were exposed to NMs and not to soluble chemicals (Bortezomib), as was the case in the Lenz et al. study, which may explain why we did not observe a correlation of the results between the ALI and submerged conditions by normalizing the LOAELs to the number of cells exposed. Indeed, poorly soluble NMs are toxic through surface reactivity [64]. Moreover, in contrast to chemicals which are highly soluble, the doses of poorly soluble NMs are heterogeneously distributed on cells, especially in the complex co-culture model presenting an uneven surface. For this reason we thought that expressing the doses in μg/cm^2^ rather than in μg/cells may be better to describe our results. Nevertheless, further investigations are still needed to assess if μg/cm^2^ is a better dose metric for poorly soluble NMs.

To conclude, the lower LOAELs observed in the ALI conditions were not due to higher basolateral cytokine secretion by the cells or because of different cell numbers. The differences were unlikely caused by NM-cytokine interactions in the submerged conditions, as the cytokines were dosed separately in the apical and basolateral sides to limit this bias (for exposure in inserts only). For these reasons, we hypothesized that the differences may have been due to a higher sensitivity of the cells to the NMs or at least to particles at the ALI. However, it was not possible to conclude this with certainty. Indeed, we could not totally exclude that uncertainties regarding the measurements of the deposited doses on cells, especially in submerged conditions where no direct dosage was performed, could explain the differences in LOAELs, of at least a 3 fold order of magnitude, observed between ALI and submerged exposure in inserts. Thus, more accurate dosimetry should be considered in the future to provide better comparisons.

After exposure to NMs, direct comparisons between ALI and submerged results have been performed in other studies, leading to different conclusions. As was observed in our study, Lenz et al. observed effects at lower doses when cells were exposed to ZnO NPs at the ALI [51]. Using ZnO NM, Xie et al. observed toxic effects at “doses that are in the same order of magnitude” [39]. Using SiO_2_-50 and Ag NM respectively, Panas et al. [38] and Herzog et al. [52] concluded that the nanoparticles were less toxic when deposited at the ALI. In these studies, different NMs were used, which could explain the discrepancy between the conclusions. For example, some NMs have the ability to release ions in suspension [65] and it was shown that both ions and particles are able to cause toxic effects on cells [66]. Thus in some cases, the differences in toxic effects observed between ALI and submerged exposures can be attributed to an increase in the proportion of ions released in the submerged conditions [52]. Differences in cellular models, methodologies used to expose the cells to aerosols or suspensions and in methods employed to characterize the NM deposition on the cells are additional factors that could also explain the different conclusions.

Xie et al. [39] used the same method to prepare the NM suspensions to generate aerosol and submerged exposures. In this study, FBS was added to the suspension, which could induce the formation of a medium specific corona at the ALI. This might explain why they observed similar effects between the ALI and submerged exposures. To improve the deposited dose in their system, Panas et al. [38] raised the aerosol flow rate to 100 mL/min at the cellular level. As an increase of the flow rate can lead to a decrease in cell viability, they added 100 μL of PBS on the cell surface, which can lead to a decrease in the cell surface directly exposed at the ALI. Moreover in some studies, the real mass deposited on the cells after exposure to suspensions was reached after 24 h of exposure, which can create uncertainties towards the conclusions.

In conclusion, while there is a general trend towards higher sensitivity of ALI as compared to submerged exposure conditions, relatively large uncertainties in the dose estimates for submerged conditions (due to uncertainties in volume median diameter and effective density) render this result uncertain. More advanced dosimetry methods as currently available are required to resolve this issue with certainty.

#### Importance of the dose rate

In our study, the final deposited doses were reached either within 3 h in the inserts, in ALI and submerged conditions or within 24 h in the plates. We observed significant effects at lower doses in the inserts than in the plates, in the submerged conditions (Table 5). Thus, as shown recently in vivo [67], it also seems important to consider the dose delivery rate when assessing NM toxicity in vitro.

## Conclusions

The deposition of NMs on cells via aerosol exposure was validated in our system. We showed that the maximum deposited doses achieved were of about 3 μg/cm^2^ for a 3 h-exposure at the ALI. These doses were low compared to those reached in submerged exposures, but were sufficient to observe biological adverse effects (inflammation, cytotoxicity and oxidative stress) 24 h after exposure. Thus, we hypothesize that our model can be used to assess the toxicity of other metallic and insoluble NMs. Furthermore, biological adverse effects were observed in A549 + THP-1 co-cultures but not in A549 monocultures, indicating a higher sensitivity of the coculture model. This underlines the importance of the cellular model used and offers the possibility to deal with low deposition doses by using more sensitive and physiologic cellular models.

To compare the ALI results to those obtained in classic submerged experiments, we exposed the co-culture models to suspensions of NMs and assessed for biological adverse effects (inflammation, cytotoxicity and oxidative stress). Based on the quantified or estimated deposited doses on the cells, we performed direct comparisons of the results between the different exposure methods used. We observed adverse effects at lower deposited doses after exposure at the ALI to aerosols of NMs than in submerged conditions to suspensions of NMs. Furthermore, comparing submerged exposures in inserts and plates at the same dose of NMs, we showed that the biological effects observed were dependent on the timing of the dose delivery. We provided a ranking of the NMs according to the biological adverse effects observed and these were ranked similarly whatever the exposure method used. Thus, despite the differences in levels of biological adverse effects observed, we showed that the two in vitro methods provided reliable results in the assessment of potential biological adverse effects and the ranking of poorly soluble and metallic NMs. Future studies should examine more precisely why biological effects were observed at lower deposited dose at the ALI in our study. Studies with accurate dosimetry are still necessary to confirm if differences in sensitivity exist when cells are exposed at the ALI to poorly soluble NMs. It would be also interesting to determine the influence of surfactant and to assess the importance of the corona surrounding NMs, both at the ALI and in submerged conditions.

## Methods

### Nanomaterials

The nano-TiO_2_ NM105 (AEROXIDE® TiO_2_ P25) was obtained from Evonik Industries. The nanos TiO_2_ NM100 and NM101 and nano-CeO_2_ NM212 were obtained from the Joint research council (JRC). The TiO_2_ and CeO_2_ physicochemical properties (Table 1) were well characterized by the Joint research council (JRC) [68, 69]. The endotoxin levels of the NMs were tested by partners of the European project NANoREG. They were below the limit of detection (data not shown).

### Cell cultures

The human type II alveolar epithelial cell line A549 and the human alveolar monocyte cell line THP-1 were obtained from our partners of the NANoREG project (from BAUA and GAIKER, respectively). Both cell lines were cultivated in RPMI 1640 medium (Gibco, 61870), supplemented with 10 % Fetal Bovine Serum (FBS) (Gibco, 15070) and 1 % penicillin-streptomycin (Gibco, 15070) (culture medium) at 37 °C in a humidified atmosphere containing 5 % CO_2_ (Sanyo-18AIC). A549 and THP-1 cells were seeded in 75 cm^2^ tissue culture flasks (Falcon, 353136), with 700 000 A549 cells/flask and 3 000 000 THP-1 cells/flask. At 90 % confluency, A549 cells were trypsinized (Gibco, 25300), and seeded into 24-well plates (Falcon, 353047) with 50 000 cells/well, (0.5 mL of culture medium/well) for submerged exposures in plates or seeded in 6 well plates inserts (4.67 cm^2^ of diameter, 0.4 μm pore size, Costar, 3450) with 80 000 cells/insert (1 mL of culture medium at the apical side and 2 mL at the basal side/insert) for aerosol exposures and submerged exposures in inserts. For the co-cultures, THP-1 cells were differentiated into mature macrophage-like cells in culture flasks with 300 ng/mL of Phorbol Myristate Acetate (PMA) (Sigma-Aldrich, P1585) for 24 h and seeded on the A549 cells 24 h before exposure, at a ratio of one THP-1 cell to ten A549 cells. To calculate the number of A549 cells at the exposure time, cells were grown for 5 days in dedicated inserts (at the ALI and in submerged conditions) or for 72 h in plates, were trypsinized and then counted. To trypsinize cells in inserts, trypsin was added at the apical and basal side to promote cell detachment.

### Exposure at the ALI to aerosols

For monoculture exposures, A549 cells were grown for 96 h until confluence. The culture medium at the apical side of the cells was then retrieved to adapt the cells to the ALI for 20 h before exposure. For co-culture exposures, A549 cells were grown for 96 h until confluence. In the meantime, THP-1 cells were differentiated into mature macrophage-like cells. One day prior exposure, the differentiated THP-1 cells were washed, trypsinized (Gibco, 25200), centrifuged and seeded on the A549 cells with a ratio of one THP-1 cell for ten A549 cells. Four hours after seeding, the culture medium at the apical side of the cells was removed to adapt the co-cultures to the ALI for 20 h before exposure. Five days after A549 seeding, both cell models were exposed for 3 h at the ALI. Just after exposure cells were placed in new plates (Costar, 3516) with fresh culture medium containing 10 % FBS at the basal side and kept at the ALI for 21 h in the incubator. At every step, the condition of the cells was checked carefully by optical microscopy.

#### Aerosol exposure system

A system using VitroCell® devices was set up to expose mono or co-cultures to aerosols of NMs (Fig. 2). The system was composed of two chambers (VitroCell®, 6/4 and 6/3 CF Stainless cultivation base modules) in which cells seeded in inserts were exposed at the ALI to aerosols of NMs or to filtered air. The NM exposure chamber contained 4 wells. Three wells were dedicated to expose cells grown in inserts to aerosols of NMs, and one to assess the real-time deposition of the NMs on a Quartz Cristal Microbalance (QCM) (VitroCell®). The QCM was used to check if the system was running properly and to quantify the NM deposition on the inserts. The air exposure chamber contained three wells in which cells grown in inserts were exposed to filtered air containing 90 % humidity. Culture medium supplemented with 25 mM HEPES (Gibco, 156030-056) was individually supplied to each basolateral compartment of the inserts to maintain the cells at the ALI during exposure. The chambers were connected to a water bath to maintain the cells at 37 °C.

To generate the aerosols, suspensions of 1, 5 and 10 g/L of NM were prepared in Milli-Q water, and sonicated for 5 min in an ultrasonic bath (Bioblock, Leo-80). A nebulizer (Palas, AGK 2000) supplied with filtered air (Norgren, SPGB/BMR/28262) was used at a flow rate of 5 L/min, to aerosolize the suspension. A dryer composed of silica gel (Roth, P077) was used to prevent condensation into the system, maintaining the relative humidity above 90 %. To expose the cells, a vacuum pump (KnF, N840FT-18) and flow calibration valve (VitroCell®) were used to suck TiO_2_ and CeO_2_ aerosols or air into the VitroCell® chambers at a 5 mL/min flow rate.

#### Characterization of aerosols and NM deposition on cells

Mass concentrations of the NMs 105, 100, 101 and 212 in the aerosols were measured by a gravimetric method. Briefly, quartz microfiber filters (Whatman, 1851-037) were dried for 24 h in a dessicator and weighed before exposure. Samplings of the aerosols were performed in triplicates at 4 L/min for at least 10 min using a sampling pump (Casella, Apex personal air sampler). After exposure, the filters were dried for 24 h and weighed. The mass concentration was calculated according to the mass of NM weighed and the sampling volume. A Scanning Mobility Particle Sizer SMPS, (Differential Mobility Analyser (DMA) (Grimm, L-DMA 5400) with Condensation Particle Counter (CPC) (Grimm, 5.416) and X-Ray neutralizer (TSI, 3087), and an optic counter (OPC) (Grimm, 1.109) were used to measure the size distribution of the particles in the aerosol. Particles ranging from 10 nm to 30 μm of diameter were measured. According to the number size distribution measurements, the GMDs of the aerosols (equivalent to count median diameters) were calculated. VMDs were also calculated assuming perfect spherical geometry of the NMs in the aerosol for the conversion from number size distributions to volume size distributions. This assumption was made based on the observations of spherical NM agglomerates deposited at the apical side of the inserts (Fig. 3b). The effective densities of the aerosols were then calculated by dividing the aerosol concentration, measured by gravimetry, by the total aerosol volume concentration, calculated by SMPS and OPC.

The deposition of the NMs on the cells after 3 h of exposure was also characterized. QCM and ICP-MS measurements were performed to measure the mass of NMs deposited on cells. For ICP-MS analysis, A549 cells at confluence were exposed for 3 h to aerosols of NMs. At the end of the exposure period, the insert membranes were directly cut with a scalpel and kept in tubes (Dutsher, 030402) at -20 °C before analysis. Samples were mineralized to perform the analysis. According to the deposited masses measured, the deposition efficiencies on the cells were calculated. To do this, the theoretical deposited masses were calculated assuming 100 % of deposition on the cells, by dividing the mass concentration of the aerosols by the aerosol volume passing through the exposure chambers within 3 h. The deposition efficiencies were calculated for each NM and concentration used by dividing the mass measured (by QCM or ICP-MS) by the theoretical deposited mass. Transmission Electron Microscopy (TEM) grids (Agar scientific, Quantifoil S143-3) were used to assess the shapes, sizes, and distributions of the NMs deposited. TEM grids were deposited on the apical side of the inserts, exposed for 3 h to aerosols of NMs and then analyzed by TEM.

### Submerged exposure to NM suspensions

To expose co-cultures to suspensions, THP-1 cells were first differentiated into mature macrophage-like cells with 300 ng/mL of PMA in culture flasks for 24 h. Differentiated THP-1 cells were trypsinized, washed, centrifuged and seeded on A549 cells 20 h before exposure, to achieve a ratio of ten A549 cells to one THP-1 cell. In inserts, 5 days after A549 seeding, the co-cultures were exposed at the apical side to NM suspensions in culture medium containing 10 % FBS with concentrations ranging from 23 to 545 μg/mL depending on the NMs used, to achieve deposited doses of 1, 3 and 10 μg/cm^2^ after 3 h of exposure (Table 4). After 3 h of exposure, the apical NM suspensions and the basal medium were removed carefully, to ensure that all the particles deposited remained on the cell surface and were replaced by fresh culture medium containing 10 % FBS. Cells were kept for the remaining 21 h in the incubator with NMs deposited on their surface. In plates, 72 h after A549 seeding, cells were exposed for 24 h to suspensions of NMs in culture medium containing 10 % FBS, with concentrations ranging from 4 μg/mL to 200 μg/mL (equivalent to about 1 μg/cm^2^ to 20 μg/cm^2^ deposition, depending on the deposition ratio calculated) (Table 4).

#### Characterization of suspensions and NM deposition on cells

Stock suspensions of NMs were prepared in Milli-Q water at 2.56 mg/mL. Suspensions were dispersed with a sonicator equipped with a cup horn (QSONICA, Q700), at maximum amplitude, at a frequency of 2 times 1 min with a pause of 1 min between. The cup horn indicated the total energy delivered to the volume of water in the cup (1 L) and to the sample (40 000 J). We also estimated the energy delivered to the sample experimentally (97 J), as described in the Additional file 1. To expose the cells, sonicated suspensions were first diluted in culture medium and successive dilutions were performed to achieve the desired concentrations. For each NM, DLS measurements were performed (Malvern, Zetasizer Nano S) on stock and 0.4 mg/mL suspensions to measure the hydrodynamic diameter and assess the size distribution of the particles in suspension. The effective densities of each NM were measured in suspension according to the VCM method developed by Deloid and coworkers [56].

The ISDD model [30] was used to estimate the deposited fraction on cells after 3 h of exposure in inserts or 24 h of exposure in plates. The primary particle diameters, the primary densities of the powders, the hydrodynamic diameters, the effective densities of the NMs measured in suspensions, the height (0.214 cm for inserts, 0.25 cm for plates), the temperature (37 °C), the density (1.00 g/mL), the viscosity (0.00074 Pa.s) and the nominal NM concentrations (4 to 547 μg/mL) of the medium were used as input parameters. For results interpretation, nominal NM concentrations expressed in μg/cm^2^ were adjusted according to the estimated deposited fraction.

### Alamar blue® assay (cell functionality)

After 24 h of exposure to NMs, an Alamar blue® assay was performed to measure the metabolic activity of the cells exposed to aerosols or suspensions. The culture medium was retrieved and the cells were washed with HBSS (Gibco, 140025). Some washing liquids (1 mL/sample) from aerosol exposures were also kept for analysis. Cells were then incubated at 37 °C, in 5 % CO_2_ for 1 h submerged with 0.5 mL (in plates) or 1 mL (in inserts) of Alamar blue® solution (Invitrogen, prestoblue A13261) diluted to 10 % in culture medium. After 1 h of incubation, 100 μl of metabolized Alamar blue® was transferred in a 96 well plate (Falcon, 353072) and the fluorescence was read (excitation: 555 nm, emission: 585 nm) using a spectrophotometer (TECAN, infinite 2000). The values of each sample were expressed in percentage of cell functionality compared to control. Cells exposed to clean air at the ALI served as controls for the aerosol exposures; cells exposed to culture medium served as controls for suspension exposures.

### LDH assay (cell integrity)

LDH releases in apical or basal sides were measured in culture media retrieved after 24 h of exposure to suspensions and aerosols and kept at 4 °C until analysis. Culture media, retrieved at the apical sides, were centrifuged for 5 min at 13 000 G and 4 °C to remove the NMs. A commercially available kit (Promega, CytoTox-ONE Homogeneous Membrane Integrity assay) was used according to the supplier manual. The fluorescence was measured (excitation: 550 nm, emission: 585 nm) using a spectrophotmeter (TECAN infinite 2000). The values of each sample were expressed in function of the maximum LDH release by cells, in percentage of cell integrity compared to control. To measure the maximum LDH release, cells were lysed for 24 h using a 0,1X solution of triton (Sigma-Aldrich, T8787). For exposure to suspensions in inserts, results from the apical and basal sides were pooled to evaluate the cell integrity.

### Dichlorofluorescein (DCF) assay (Intracellular ROS)

After performing Alamar blue® assays, the cells were washed with PBS (Gibco, 10270). Afterwards, the cells were incubated at 37 °C, in 5 % CO_2_ with 10 μM of a 5-(and-6)-chloromethyl-2′,7′-dichlorodihydrofluorescein diacetate (CM-H2DCFDA) probe (Life technologies, C6827) in PBS (0.5 mL/well or insert) for 35 min. After 30 min of incubation, the probe was removed in some wells, 1 mM of H_2_O_2_ in PBS was added and the cells were incubated for 5 min to serve as positive controls. After incubation, the cells were washed with PBS and incubated for 5 min in 90 % Dimethyl Sulfoxide (DMSO) (Sigma-Aldrich, D2438) in PBS (0.5 mL/well or insert). Then the cells were scraped using a scraper (TPP, 99002), the well or inserts contents were recovered in tubes (Eppendorf, 3810X) and the tubes were centrifuged at 10 000 G, at 4 °C, for 5 min to eliminate dead cells and remove remnants particles. The tube contents were transferred into black 96 well plates (150 μL/well) (Greiner Bio-one, 655076) and the fluorescence of the samples was read (excitation: 488 nm, emission: 530 nm) using a spectrophotometer (TECAN, infinite 2000). The values of each sample were expressed in percentage of intracellular ROS compared to their respective control.

### Cytokine and chemokine quantification by ELISA

Pro-inflammatory mediator levels were measured in culture media or washing liquids retrieved after 24 h of exposure from aerosol and submerged exposures and kept at -80 °C until analysis. Before freezing, culture media and washing liquids were retrieved from the apical side of submerged and aerosol exposures and centrifuged for 5 min at 13 000 G at 4 °C to remove the nanoparticles. Il-1β, IL-6, IL-8 and TNF-α release was measured using a commercially available ELISA multiplex kit (Mesoscale discovery, K15025B) and a multiplex reader (Mesoscale discovery, Sector Imager 24000), according to the supplier recommendations. The values of each sample were expressed in percentage of cytokine levels compared to the respective control. Cells stimulated for 21 h to a concentration of 20 μg/mL of LPS (Sigma-Aldrich, L2880) (at the basal side for exposures in inserts), were used as positive control.

### Interactions between the NMs and the LDH assay

Potential interactions between LDH and NMs were assessed. 96 well plates (Falcon, 353072) were incubated under cell-free conditions for 24 h with suspensions of 0, 100, 400 μg/mL of TiO_2_ and CeO_2_ in presence of 0275 UI/mL of LDH standard (Roche, 10127876001). After 24 h of incubation, supernatants were retrieved, centrifuged for 5 min at 13 000 G at 4 °C to remove the nanoparticles and the LDH assay was performed.

### Interactions between the NMs and the cytokines

Potential interactions between NMs in suspension and the cytokines were studied. For suspension exposures, 96 well plates (Falcon, 353072) were incubated under cell-free conditions for 24 h with suspensions of 0, 100, 400 μg/mL of TiO_2_ and CeO_2_ in presence of 1250 pg/mL of IL-1β, IL-6, IL-8 and TNF-α. After 24 h of incubation, the supernatants were retrieved and centrifuged for 5 min at 13 000 G at 4 °C to remove the nanoparticles. IL-1β, IL-6, IL-8 and TNF-α were measured by ELISA multiplex (Mesoscale discovery, K15025B) in the supernatants.

### Statistical analysis

All the data was expressed as mean ± standard deviation (SD) from three independent experiments performed in triplicates. The statistical analysis was performed using Graphpad Prism 5.0 (GraphPad Software Inc., San Diego, CA). Shapiro-wilk’s and Bartlett’s tests were used to assess the data normality and the variance equality, respectively. Because variances weren’t equal, results were analyzed by the non-parametric Kruskal-Wallis test followed by Dunn’s post-hoc test to compare the different treated groups to the controls. Cells exposed to air at the ALI served as control for aerosol exposures. Cells exposed to culture medium served as control for suspension exposures. For alamar blue and LDH assays, values from cells exposed to NMs or control and from cells kept in the incubator (for ALI exposure only) were included in the statistical analysis. For the DCF assay, values from the cells exposed to NMs or to control air and from cells exposed to 1 mmol H_2_O_2_ were included in the statistical analysis. For ELISA assays, values from cells exposed to NMs or control and from cells stimulated with 20 μg/mL of LPS were included in statistical analysis. To assess the level of pro-inflammatory mediators at the ALI and in submerged conditions after stimulation with LPS, a two-way Anova followed by Bonferroni post-hoc test was performed to compare treated groups to controls or to compare ALI and submerged exposure. In all the analysis, *p*-values < 0.05 were considered significant.

## Additional file

Additional file 1: Figure S1. Mass size distribution of TiO_2_ and CeO_2_ aerosols. **Figure S2.** Functionality and integrity of mono and co-cultures exposed at the ALI. **Figure S3** Number size distributions of TiO_2_ (NM105, 101, 100) and CeO_2_ (NM212) NM in suspensions used to expose cells. **Figure S4.** Interactions between the NMs and LDH or with cytokines in suspensions. **Table S1.** Alamar blue results expressed in percentage of functionality compared to control. **Table S2.** LDH results expressed in percentage of integrity compared to control. **Table S3.** DCF results expressed in percentage of intracellular ROS compared to control. **Table S4.** IL-1β results expressed in percentage compared to control. **Table S5.** IL-6 results expressed in percentage compared to control. **Table S6.** IL-8 results expressed in percentage compared to control. **Table S7** TNF-α results expressed in percentage compared to control. **Table S8.** IL-1β levels at the basal and apical sides after stimulation with LPS (20 μg/mL). **Table S9.** IL-6 levels at the basal and apical sides after stimulation with LPS (20 μg/mL). **Table S10.** IL-8 levels at the basal and apical sides after stimulation with LPS (20 μg/mL). **Table S11** TNF-α levels at the basal and apical sides after stimulation with LPS (20 μg/mL). (DOCX 670 kb)

## Abbreviations

ALI: Air-liquid interface
DLS: Dynamic Light Scattering
DMSO: Dimethyl Sulfoxide
FBS: Fetal Bovine Serum
ICP-MS: Inductively Coupled Plasma—Mass Spectrometry
ISDD: in vitro sedimentation diffusion and dosimetry
LDH: Lactate deshydrogenase
LPS: Lipopolysaccharides
NM(s): Nanomaterial(s)
OPC: Optic counter
PMA: Phorbol Myristate Acetate
QCM: Quartz Cristal Microbalances
ROS: Reactive oxygen species
SD: Standard deviation
SMPS: Scanning Mobility Particle Sizer
TEM: Transmission Electron Microscopy
VCM: Volumetric Centrifugation Method
