# Supplementary material for: Air-liquid interface exposure to aerosols of poorly soluble nanomaterials induces different biological activation levels compared to exposure to suspensions
Source: Part Fibre Toxicol. 2016 Nov 3;13:58. doi: 10.1186/s12989-016-0171-3 (PMC5137211; doi:10.1186/s12989-016-0171-3)
Supplement: Additional file 1: Figure S1. — Mass size distribution of TiO2 and CeO2 aerosols. Figure S2. Functionality and integrity of mono and co-cultures exposed at the ALI. Figure S3 Number size distributions of TiO2 (NM105, 101, 100) and CeO2 (NM212) NM in suspensions used to expose cells. Figure S4. Interactions between the NMs and LDH or with cytokines in suspensions. Table S1. Alamar blue results expressed in percentage of functionality compared to control. Table S2. LDH results expressed in percentage of integrity compared to control. Table S3. DCF results expressed in percentage of intracellular ROS compared to control. Table S4. IL-1β results expressed in percentage compared to control. Table S5. IL-6 results expressed in percentage compared to control. Table S6. IL-8 results expressed in percentage compared to control. Table S7 TNF-α results expressed in percentage compared to control. Table S8. IL-1β levels at the basal and apical sides after stimulation with LPS (20 μg/mL). Table S9. IL-6 levels at the basal and apical sides after stimulation with LPS (20 μg/mL). Table S10. IL-8 levels at the basal and apical sides after stimulation with LPS (20 μg/mL). Table S11 TNF-α levels at the basal and apical sides after stimulation with LPS (20 μg/mL). (DOCX 670 kb) [file 12989_2016_171_MOESM1_ESM.docx]

**Additional file**

**Figure S1.** Mass size distribution of TiO_2_ and CeO_2_ aerosols.

**Figure S2.** Functionality and integrity of mono and co-cultures exposed at the ALI.

**Figure S3.** Size distribution of TiO_2_ (NM105, 101, 100) and CeO_2_ (NM212) NMs in suspensions used to expose cells.

**Estimation of the energy delivered to the NM suspensions using a sonicator equipped with a cup horn.**

**Interaction between NMs and assays**

**Figure S4.** Interactions between the NMs and LDH or with cytokines in suspensions.

**Tables of results**

**Table S1.** Alamar blue results expressed in percentage of functionality compared to control.

**Table S2.** LDH results expressed in percentage of integrity compared to control.

**Table S3.** DCF results expressed in percentage of intracellular ROS compared to control.

**Table S4.** IL-1b results expressed in percentage compared to control.

**Table S5.** IL-6 results expressed in percentage compared to control.

**Table S6.** IL-8 results expressed in percentage compared to control.

**Table S7.** TNF-α results expressed in percentage compared to control.

**Table S8.** IL-1β levels at the basal and apical sides after stimulation with LPS (20 µg/mL).

**Table S9.** IL-6 levels at the basal and apical sides after stimulation with LPS (20 µg/mL).

**Table S10.** IL-8 levels at the basal and apical sides after stimulation with LPS (20 µg/mL).

**Table S11.** TNF-α levels at the basal and apical sides after stimulation with LPS (20 µg/mL).

**Figure S1. Mass size distribution of TiO_2_ and CeO_2_ aerosols.**

Aerosols were generated by nebulization of suspensions of TiO_2_ (NM105, 101, 100) and CeO_2_ (NM212) at concentrations of 1 g/L (light grey), 5 g/L (dark grey) and 10 g/L (black). The mass size distribution of the NMs in aerosols was assessed using a Scanning Mobility Particle Sizer (SMPS) and an Optic Counter (OPC), measuring particles ranging from 10 to 1095 nm and 300 to 34 000 nm, respectively.


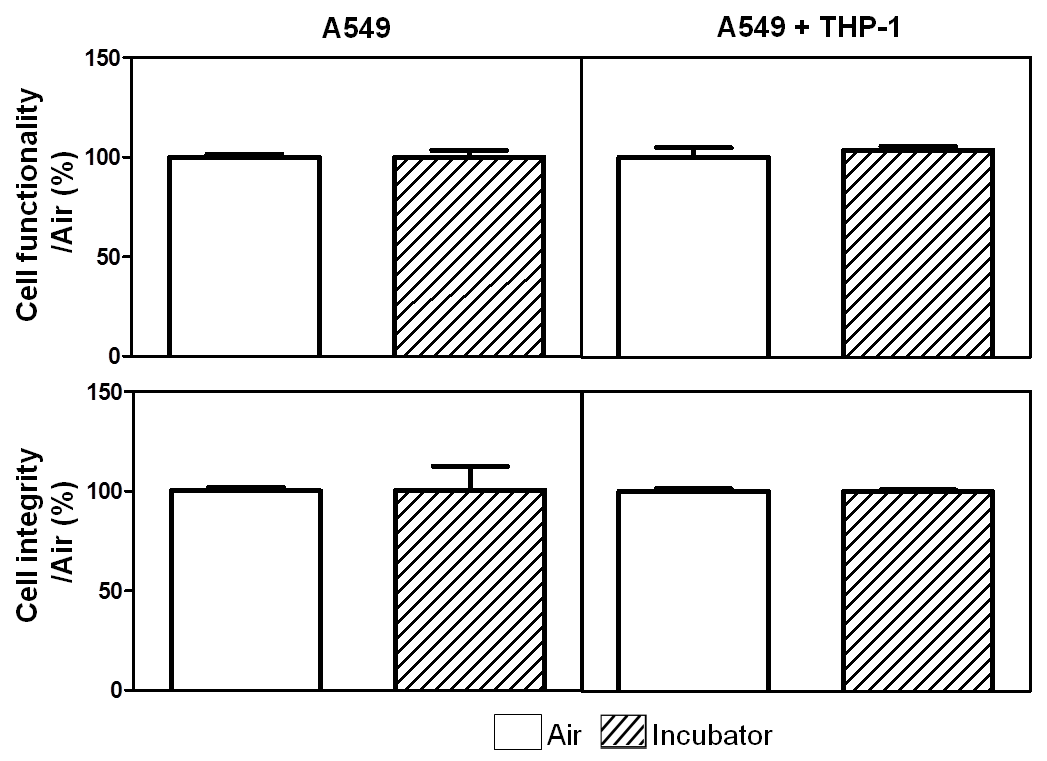


**Figure S2. Functionality and integrity of mono and co-cultures exposed at the ALI.**

Cells were exposed for 3h to air in a VitroCell^®^ system and then kept at the ALI in the incubator for the remaining 21h (Air) or kept at the ALI in the incubator during 24h (Incubator). Alamar blue^®^ and LDH assays were performed to assess functionality and integrity of the cells, respectively. Data represents the mean ± SD of three independent experiments. A Kruskal-Wallis test followed by Dunn’s post-hoc test were performed to compare treated groups to control (*p<0.05; **p<0.01; ***p<0.001).


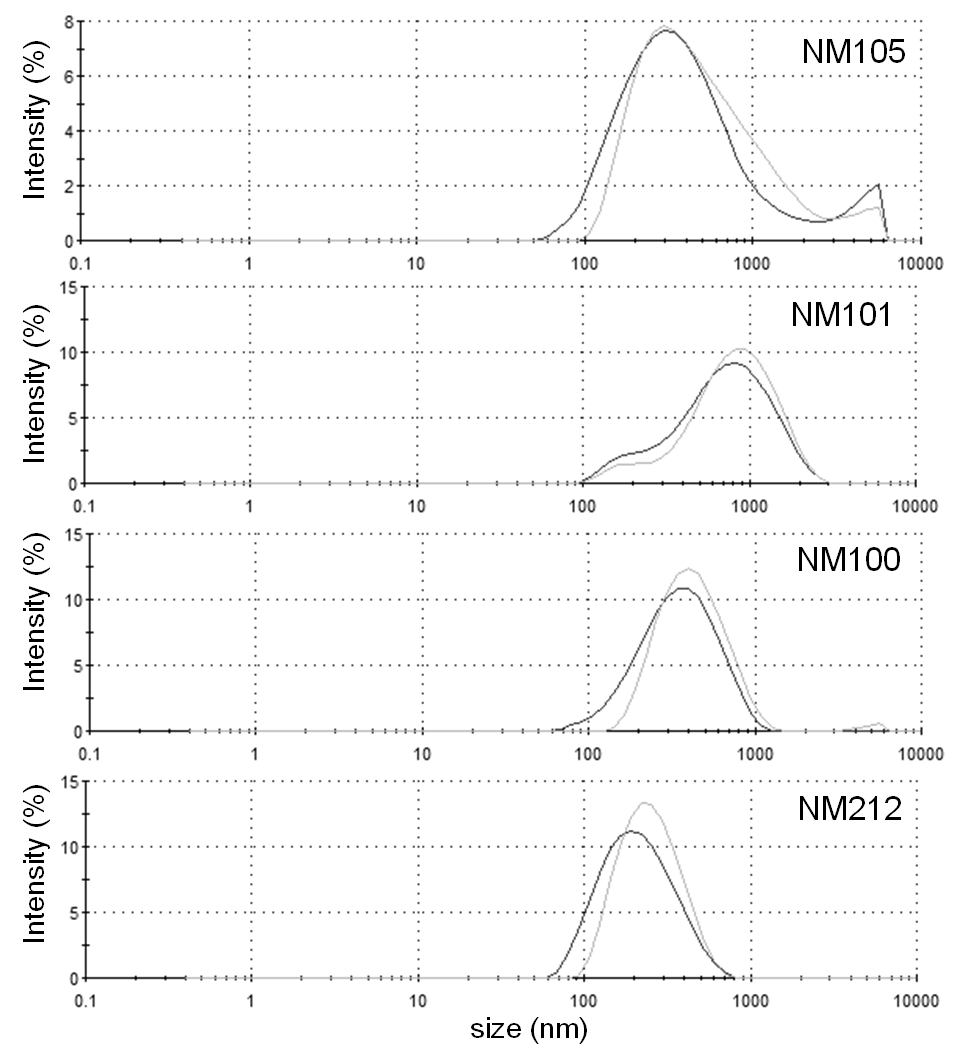


**Figure S3. Size distribution of TiO_2_ (NM105, 101, 100) and CeO_2_ (NM212) in suspensions used to expose cells.** DLS measurements were performed after sonication in stock suspensions (2.56 mg/mL in milli-Q water) (dark grey) and after dilution in 0.4 mg/mL suspensions in culture medium (light grey).

**Estimation of the energy delivered to the NM suspensions using a sonicator equipped with a cup horn.**

**Methods**

To estimate the energy delivered to the NM suspensions using a cup horn sonicator, we measured the cooling rate of a sample of Milli-Q water, in the cup of a cup horn connected to a 4°C ice bath. After reaching 19°C, the temperature of the sample was recorded every 15 s for 1 min (n=3) in the absence of or with sonication (1 min, amplitude 100). We assumed that a decrease in the cooling rate was caused by the energy delivered to the sample through sonication. Once the cooling rate became linear (at around 14°C), we measured the difference in temperature between sonicated and non-sonicated samples and plotted the differential cooling rate on a graph and measured the slope. We then used the equation proposed by Taurozzi et al. (2011) [[1](#_ENREF_1)] to measure the power delivered to the sample:

Power = differential cooling rate slope (K/s) * mass of liquid (g) * specific heat of the liquid (4.18 J/g*K for water)

**Results**

The differential cooling rate curve was plotted and we obtained a linear fit ( R² = 0.99). The slope of the curve was determined (0.0322 K/s). According to the mass of liquid in the sample (6 g) and the specific heat of the liquid (4.18 J/g*K), the power delivered to the sample was calculated (0.81 J/s). Then, the total energy delivered to the sample (97 J) was calculated according to the time of sonication (120 s).

**Interaction between NMs and assays**

**Results**

**Interactions between NMs and LDH**

After 24h of incubation under cell-free conditions with suspensions at concentrations of 0, 100, 400 µg/mL of TiO_2_ or CeO_2_ and in presence of 0.275 UI/mL of LDH, LDH levels were assessed in the supernatants. No significant differences in LDH levels were observed (0 µg/mL), compared to control (Figure S3a).

**Interactions between NMs and cytokines**

After 24h of incubation in cell-free conditions with suspensions at concentrations of 0, 100, 400 µg/mL of TiO_2_ (NMs 105, 101, 100) or CeO_2_ (NM212) and in the presence of 1250 pg/mL of IL-1β, IL-6, IL-8 and TNF-α, cytokine concentrations were assessed by ELISA (Figure S3b). Interactions between NMs and cytokines were observed with the three NMs but not with the NM CeO_2_. At 1250 pg/mL and in the presence of 100 µg/mL of TiO_2_ NM101, significant decreases in IL-8 concentration were observed, compared to control. In the presence of 400 µg/mL of NM105, significant increases in IL-1β and significant decreases in IL-8 and TNF-α concentrations were observed. In the presence of 400 µg/mL of NM101, significant decreases in IL-6, IL-8 and TNF-α concentrations were observed. In the presence of 400 µg/mL of NM100, significant increases in IL-1β and significant decreases in IL-8 concentrations were observed.


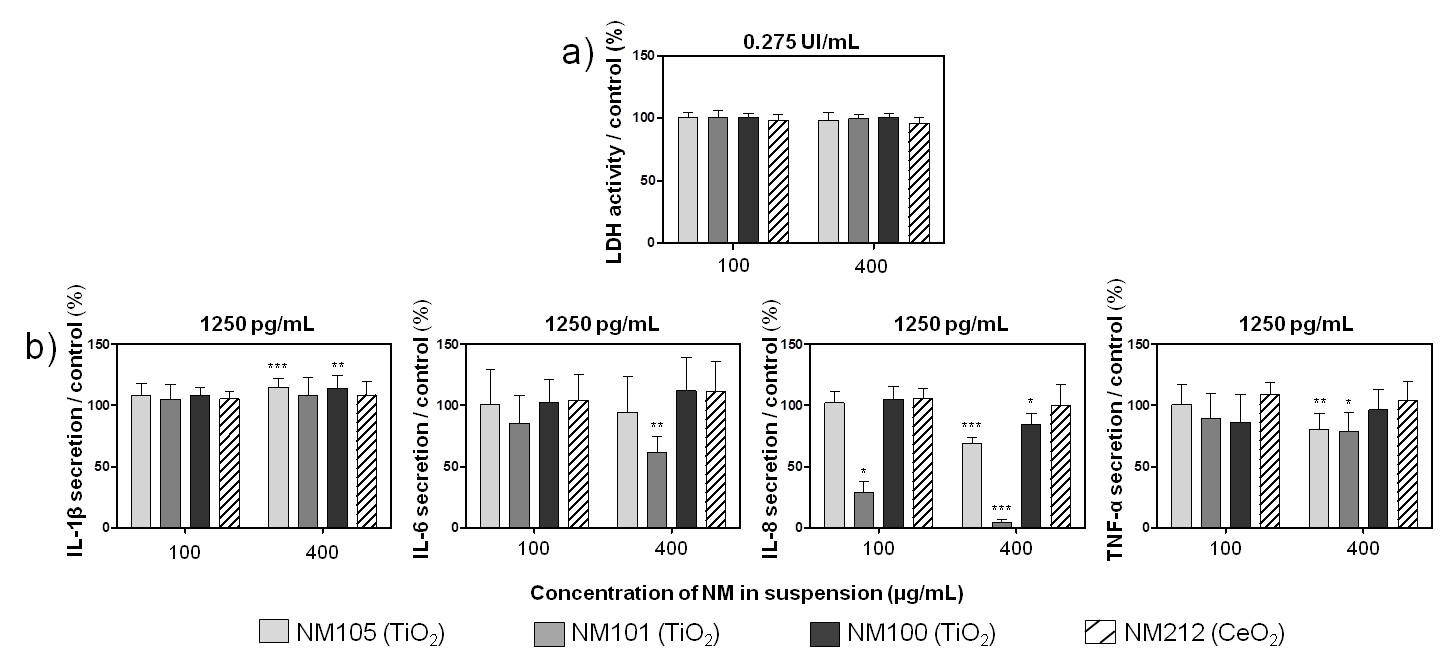


**Figure S4. Interactions between NMs and LDH (a) or NMs and cytokines (b) in suspensions.** Plates were incubated in cell-free conditions for 24h with suspensions at concentrations of 0, 100, 400 µg/mL of TiO_2_ (NMs 105, 101 and 100) and CeO_2_ (NM212), in the presence of 0.275 UI/mL of LDH (a) or in the presence of 1250 pg/mL of IL-1β, IL-6, IL-8 and TNF-α (b). LDH and cytokine levels were assessed in the supernatants, after centrifugation for 5 min at 13 000 G. Data represents the mean ± SD of three independent experiments. A Kruskal-Wallis test followed by Dunn’s post-hoc test were performed to compare treated groups to control (*p<0.05; **p<0.01; ***p<0.001).

**Tables of results**

|  |  |  | **NM105** | | | **NM101** | | | **NM100** | | | **NM212** | | |
| --- | --- | --- | --- | --- | --- | --- | --- | --- | --- | --- | --- | --- | --- | --- |
| Exposure method | Cell model | Dose | Mean | SD | n | Mean | SD | n | Mean | SD | n | Mean | SD | n |
| ALI | A549 | 0 | 100.00 | 1.43 | 8 | 100.00 | 1.23 | 9 | 100.00 | 1.83 | 9 | 100.00 | 1.03 | 9 |
|  |  | 3 (µg/cm²) | 97.87 | 5.00 | 8 | 100.40 | 1.35 | 9 | 93.42 | 5.77 | 9 | 99.72 | 1.04 | 9 |
|  |  | incubator | 99.94 | 3.51 | 9 | 100.40 | 1.35 | 9 | 102.10 | 2.33 | 9 | 100.50 | 2.07 | 9 |
|  | A549 + THP-1 | 0 | 100.00 | 4.42 | 9 | 100.00 | 4.65 | 9 | 100.00 | 2.02 | 9 | 100.00 | 3.05 | 9 |
|  |  | 0.1 (µg/cm²) | 105.20 | 11.56 | 9 | 103.70 | 3.99 | 9 | 98.63 | 2.27 | 9 | 100.60 | 3.13 | 9 |
|  |  | incubator | 109.60 | 8.49 | 9 | 103.40 | 3.84 | 6 | 99.96 | 2.59 | 9 | 103.20 | 4.43 | 9 |
|  |  | 0 | 100.00 | 4.90 | 9 | 100.00 | 5.79 | 9 | 100.00 | 0.46 | 9 | 100.00 | 2.40 | 9 |
|  |  | 1 (µg/cm²) | 98.20 | 4.75 | 9 | 111.40 | 12.49 | 9 | 98.70 | 4.12 | 9 | 101.80 | 1.68 | 9 |
|  |  | incubator | 103.60 | 1.96 | 9 | 111.20 | 13.35 | 9 | 101.10 | 2.00 | 9 | 103.10 | 3.44 | 9 |
|  |  | 0 | 100.00 | 5.296 | 9 | 100.00 | 8.20 | 9 | 100.00 | 5.72 | 9 | 100.00 | 3.17 | 9 |
|  |  | 3 (µg/cm²) | 97.59 | 9.44 | 9 | 105.10 | 10.48 | 9 | 103.70 | 4.45 | 9 | 96.82 | 5.00 | 9 |
|  |  | incubator | 109.80 | 12.80 | 8 | 108.70 | 7.09 | 9 | 108.70 | 9.30 | 9 | 103.90 | 3.85 | 9 |
| Submerged in inserts | A549 + THP-1 | 0 | 100.00 | 1.48 | 9 | 100.00 | 1.33 | 9 | 100.00 | 2.09 | 9 | 100.00 | 1.37 | 9 |
|  |  | 1 (µg/cm²) | 99.01 | 1.92 | 9 | 100.70 | 0.76 | 9 | 98.55 | 2.10 | 9 | 100.00 | 1.40 | 9 |
|  |  | 3 (µg/cm²) | 98.99 | 2.42 | 9 | 99.94 | 1.36 | 9 | 98.96 | 0.74 | 9 | 98.99 | 1.87 | 9 |
|  |  | 10 (µg/cm²) | 94.74** | 3.43 | 9 | 99.23 | 0.87 | 9 | 98.25 | 0.96 | 9 | 96.74** | 3.04 | 9 |
| Submerged in plates | A549 + THP-1 | 0 | 100.00 | 0.41 | 9 | 100.00 | 0.81 | 9 | 100.00 | 1.62 | 9 | 100.00 | 4.75 | 9 |
|  |  | 1 (µg/cm²) | 98.49 | 2.48 | 9 | 100.70 | 3.64 | 9 | 97.35 | 4.91 | 9 | 102.20 | 5.12 | 9 |
|  |  | 3 (µg/cm²) | 96.06* | 2.04 | 9 | 98.47 | 2.51 | 9 | 101.00 | 8.70 | 9 | 98.28 | 2.77 | 9 |
|  |  | 10 (µg/cm²) | 93.59*** | 3.10 | 9 | 97.58 | 2.55 | 9 | 97.88 | 8.52 | 9 | 95.99* | 3.81 | 9 |
|  |  | 20 (µg/cm²) | 89.86*** | 5.51 | 9 | 96.87* | 2.17 | 9 | 99.43 | 9.12 | 9 | 97.87 | 2.60 | 9 |

**Table S1. Alamar blue results expressed in percentage of functionality compared to control.**

|  |  |  | **NM105** | | | **NM101** | | | **NM100** | | | **NM212** | | |
| --- | --- | --- | --- | --- | --- | --- | --- | --- | --- | --- | --- | --- | --- | --- |
| Exposure method | Cell model | Dose | Mean | SD | n | Mean | SD | n | Mean | SD | n | Mean | SD | n |
| ALI | A549 | 0 | 100.00 | 1.78 | 8 | 100.00 | 0.592 | 9 | 100.00 | 3.43 | 9 | 100.00 | 0.38 | 9 |
|  |  | 3 (µg/cm²) | 100.90 | 2.63 | 8 | 99.95 | 0.43 | 9 | 90.04 | 14.65 | 9 | 100.3 | 0.54 | 9 |
|  |  | incubator | 100.5 | 12.04 | 9 | 99.95 | 0.43 | 9 | 100.1 | 5.33 | 9 | 99.3 | 1.58 | 9 |
|  | A549 + THP-1 | 0 | 100.00 | 1.58 | 9 | 100.00 | 1.39 | 9 | 100.00 | 0.95 | 9 | 100.00 | 1.03 | 9 |
|  |  | 0.1 (µg/cm²) | 98.86 | 3.90 | 9 | 99.85 | 2.33 | 9 | 98.60 | 2.20 | 9 | 100.3 | 0.68 | 9 |
|  |  | incubator | 103 | 2.54 | 9 | 99.05 | 5.96 | 9 | 99.39 | 3.59 | 9 | 97.07 | 5.08 | 9 |
|  |  | 0 | 100 | 1.439 | 9 | 100.00 | 1.39 | 9 | 100.00 | 0.92 | 9 | 100.00 | 1.25 | 9 |
|  |  | 1 (µg/cm²) | 97.71* | 2.821 | 9 | 99.97 | 1.24 | 9 | 99.04 | 2.33 | 9 | 103.5 | 4.59 | 9 |
|  |  | incubator | 99.89 | 0.95 | 9 | 96.65 | 7.32 | 9 | 99.80 | 3.72 | 9 | 103.40 | 5.84 | 9 |
|  |  | 0 | 100 | 2.206 | 9 | 100.00 | 2.59 | 9 | 100.00 | 6.41 | 9 | 100.00 | 1.60 | 9 |
|  |  | 3 (µg/cm²) | 88.47 | 12.87 | 9 | 99.04 | 2.33 | 9 | 96.13 | 11.55 | 9 | 96.31 | 5.93 | 9 |
|  |  | incubator | 104 | 12.34 | 8 | 100.50 | 4.61 | 9 | 97.77 | 6.12 | 9 | 101.80 | 1.50 | 9 |
| Submerged in inserts | A549 + THP-1 | 0 | 100.00 | 0.67 | 9 | 100.00 | 0.34 | 9 | 100.00 | 0.84 | 9 | 100.00 | 0.90 | 9 |
|  |  | 1 (µg/cm²) | 97.09 | 2.40 | 8 | 98.93 | 2.33 | 9 | 99.09 | 0.91 | 9 | 99.86 | 0.94 | 9 |
|  |  | 3 (µg/cm²) | 95.38** | 1.76 | 9 | 97.04* | 1.76 | 9 | 98.77 | 0.91 | 9 | 99.43 | 1.32 | 9 |
|  |  | 10 (µg/cm²) | 66.92*** | 3.79 | 9 | 88.06*** | 4.38 | 9 | 95.62*** | 2.61 | 9 | 97.9** | 1.07 | 9 |
| Submerged in plates | A549 + THP-1 | 0 | 100.00 | 0.25 | 9 | 100.00 | 0.45 | 9 | 100.00 | 0.43 | 9 | 100.00 | 0.84 | 9 |
|  |  | 1 (µg/cm²) | 100.1 | 0.75 | 9 | 99.62 | 1.37 | 9 | 99.21 | 1.30 | 9 | 99.93 | 1.05 | 9 |
|  |  | 3 (µg/cm²) | 99.66 | 0.50 | 9 | 99.2 | 0.66 | 9 | 100.5 | 0.57 | 9 | 98.58 | 1.54 | 9 |
|  |  | 10 (µg/cm²) | 96.87** | 2.22 | 9 | 97.79** | 1.04 | 9 | 99.9 | 1.06 | 9 | 98.73 | 1.64 | 9 |
|  |  | 20 (µg/cm²) | 88.68*** | 10.74 | 9 | 96.21*** | 0.72 | 9 | 99.86 | 0.35 | 9 | 98.99 | 1.71 | 9 |

**Table S2. LDH results expressed in percentage of integrity compared to control.**

|  |  |  | **NM105** | | | **NM101** | | | **NM100** | | | **NM212** | | |
| --- | --- | --- | --- | --- | --- | --- | --- | --- | --- | --- | --- | --- | --- | --- |
| Exposure method | Cell model | Dose | Mean | SD | n | Mean | SD | n | Mean | SD | n | Mean | SD | n |
| ALI | A549 | 0 | 100.00 | 3.80 | 8 | 100.00 | 2.52 | 9.00 | 100.00 | 6.88 | 9 | 100.00 | 6.10 | 9 |
|  |  | 3 (µg/cm²) | 99.8 | 12.6 | 8 | 104.20 | 2.5 | 9 | 94.5 | 10.8 | 9 | 94.2 | 3.3 | 9 |
|  |  | H_2_O_2_ | 176.2** | 16.0 | 9 | 129.8*** | 15.6 | 6 | 131.9** | 14.5 | 6 | 163.8* | 14.5 | 6 |
|  | A549 + THP-1 | 0 | 100.0 | 4.0 | 9 | 100.0 | 7.1 | 9 | 100.0 | 10.5 | 9 | 100.0 | 9.7 | 9 |
|  |  | 0.1 (µg/cm²) | 107.5 | 9.6 | 9 | 102.5 | 7.6 | 9 | 98.6 | 3.4 | 9 | 93.7 | 9.7 | 9 |
|  |  | H_2_O_2_ | 197.9*** | 22.5 | 6 | 147.3 ** | 42.1 | 6 | 157.7*** | 19.0 | 6 | 166.7** | 22.2 | 6 |
|  |  | 0 | 100.0 | 5.8 | 9 | 100.0 | 4.4 | 9 | 100.0 | 4.4 | 9 | 100.0 | 3.5 | 9 |
|  |  | 1 (µg/cm²) | 106.4 | 11.6 | 9 | 112.1 * | 8.1 | 9 | 100.0 | 4.6 | 9 | 101.0 | 7.4 | 9 |
|  |  | H_2_O_2_ | 215.4*** | 45.4 | 9 | 170.9 *** | 11.2 | 4 | 142.8** | 11.1 | 6 | 203.5** | 76.2 | 6 |
|  |  | 0 | 100.0 | 6.1 | 9 | 100.0 | 6.9 | 9 | 100.0 | 9.9 | 9 | 100.0 | 6.3 | 9 |
|  |  | 3 (µg/cm²) | 116.7 | 31.3 | 9 | 122.8** | 14.9 | 9 | 97.2 | 20.3 | 9 | 112.6 | 13.0 | 9 |
|  |  | H_2_O_2_ | 195.8** | 39.9 | 3 | 133.1** | 27.2 | 6 | 187.9** | 56.3 | 6 | 247.8*** | 125.8 | 7 |
| Submerged in inserts | A549 + THP-1 | 0 | 100.0 | 2.3 | 9 | 100.0 | 2.1 | 9 | 100.0 | 3.0 | 9 | 100.0 | 4.5 | 9 |
|  |  | 1 (µg/cm²) | 106.6 | 7.9 | 8 | 102.9 | 5.8 | 9 | 104.1 | 6.4 | 9 | 96.4 | 4.4 | 9 |
|  |  | 3 (µg/cm²) | 112.4 | 12.2 | 9 | 111.0 | 18.1 | 9 | 111.1 | 12.1 | 9 | 95.2 | 8.0 | 9 |
|  |  | 10 (µg/cm²) | 116.4* | 9.7 | 9 | 111.5 | 20.7 | 9 | 113.7* | 8.7 | 9 | 93.6 | 4.3 | 9 |
|  |  | H_2_O_2_ | 180.1*** | 11.1 | 8 | 180.1*** | 11.1 | 8 | 180.1*** | 11.1 | 8 | 180.1 | 11.1 | 8 |
| Submerged in plates | A549 + THP-1 | 0 | 100.0 | 6.4 | 9 | 100.0 | 9.9 | 9 | 100.0 | 10.5 | 9 | 100.0 | 4.6 | 9 |
|  |  | 1 (µg/cm²) | 96.2 | 11.4 | 9 | 99.5 | 13.8 | 9 | 84.4 | 11.7 | 9 | 91.9 | 10.1 | 9 |
|  |  | 3 (µg/cm²) | 113.4 | 17.6 | 9 | 111.3 | 19.7 | 9 | 90.4 | 17.0 | 9 | 96.4 | 10.2 | 9 |
|  |  | 10 (µg/cm²) | 150** | 12.5 | 9 | 112.0 | 33.2 | 9 | 94.3 | 13.2 | 9 | 98.3 | 20.6 | 9 |
|  |  | 20 (µg/cm²) | 172.2*** | 43.7 | 9 | 143.3 | 55.7 | 9 | 113.6 | 22.4 | 9 | 105.1 | 21.0 | 9 |
|  |  | H_2_O_2_ | 155.9** | 13.8 | 7 | 129.4 | 15.0 | 3 | 174.2* | 23.8 | 7 | 151.2* | 16.0 | 7 |

**Table S3. DCF results expressed in percentage of intracellular ROS compared to control.**

|  |  |  | **NM105** | | | **NM101** | | | **NM100** | | | **NM212** | | |
| --- | --- | --- | --- | --- | --- | --- | --- | --- | --- | --- | --- | --- | --- | --- |
| Exposure method | Cell model | Dose | Mean | SD | n | Mean | SD | n | Mean | SD | n | Mean | SD | n |
| ALI. secretion in Basal side | A549 | 0 | 100.00 | 8.28 | 9 | 100.00 | 20.48 | 9 | 100.00 | 20.25 | 9 | 100.00 | 9.46 | 9 |
|  |  | 3 (µg/cm²) | 106.30 | 15.01 | 9 | 93.96 | 18.06 | 9 | 103.80 | 28.36 | 9 | 99.42 | 26.82 | 9 |
|  |  | LPS | 150** | 20.28 | 6 | 139.2* | 34.65 | 6 | 142.1** | 9.78 | 6 | 155.4* | 17.33 | 5 |
|  | A549 + THP-1 | 0 | 100.00 | 9.45 | 9 | 100.00 | 16.70 | 9 | 100.00 | 37.69 | 9 | 100.00 | 64.13 | 9 |
|  |  | 0.1 (µg/cm²) | 101.50 | 10.59 | 8 | 95.13 | 47.84 | 9 | 126.30 | 30.00 | 9 | 96.76 | 33.28 | 9 |
|  |  | LPS | 828*** | 153.70 | 9 | 1037.00 | 1063.00 | 4 | 384.8* | 115.60 | 4 | 1469** | 146.80 | 6 |
|  |  | 0 | 100.00 | 20.84 | 9 | 100.00 | 33.85 | 9 | 100.00 | 48.39 | 9 | 100.00 | 29.48 | 9 |
|  |  | 1 (µg/cm²) | 269.2* | 141.90 | 9 | 535.1** | 130.80 | 9 | 159.40 | 22.41 | 9 | 105.80 | 17.58 | 9 |
|  |  | LPS | 1231*** | 571.60 | 6 | 1625*** | 776.40 | 6 | 718.5*** | 237.90 | 6 | 991.1** | 477.40 | 5 |
|  |  | 0 | 100.00 | 13.31 | 9 | 100.00 | 26.31 | 9 | 100.00 | 8.68 | 9 | 100.00 | 42.19 | 9 |
|  |  | 3 (µg/cm²) | 292.4** | 146.60 | 9 | 153.50 | 51.72 | 9 | 333.80 | 202.10 | 9 | 208.8* | 67.11 | 9 |
|  |  | LPS | 1558*** | 565.20 | 5 | 1395*** | 491.80 | 5 | 1880*** | 147.00 | 6 | 730.5*** | 149.40 | 6 |
| Submerged in inserts. secretion in Basal side | A549 + THP-1 | 0 | 100.00 | 8.87 | 9 | 100.00 | 9.09 | 9 | 100.00 | 8.90 | 9 | 100.00 | 15.48 | 9 |
|  |  | 1 (µg/cm²) | 122.30 | 26.71 | 9 | 112.00 | 25.12 | 9 | 95.54 | 10.15 | 9 | 103.70 | 10.71 | 9 |
|  |  | 3 (µg/cm²) | 148.00 | 18.84 | 9 | 123.20 | 19.94 | 9 | 94.08 | 18.07 | 9 | 93.31 | 11.89 | 9 |
|  |  | 10 (µg/cm²) | 241.4*** | 68.80 | 9 | 159.7** | 45.70 | 9 | 122.70 | 32.64 | 9 | 118.20 | 24.36 | 9 |
|  |  | LPS | 826.7*** | 454 | 9 | 826.7*** | 454 | 9 | 826.7** | 454 | 9 | 826.7*** | 454.00 | 9 |
| Submerged in inserts. secretion in Apical side | A549 + THP-1 | 0 | 100.00 | 11.21 | 9 | 100.00 | 7.41 | 9 | 100.00 | 8.20 | 9 | 100.00 | 10.64 | 9 |
|  |  | 1 (µg/cm²) | 135.90 | 31.82 | 9 | 107.40 | 10.42 | 9 | 101.60 | 15.43 | 9 | 103.70 | 11.74 | 9 |
|  |  | 3 (µg/cm²) | 163.1*** | 24.48 | 9 | 123.20 | 11.36 | 9 | 117.40 | 14.53 | 9 | 107.20 | 23.05 | 9 |
|  |  | 10 (µg/cm²) | 323.8*** | 94.47 | 9 | 194.8*** | 42.67 | 9 | 132.40 | 32.18 | 9 | 137.30 | 28.33 | 9 |
|  |  | LPS | 2443*** | 1597.00 | 9 | 2443*** | 1597.00 | 9 | 2443*** | 1597.00 | 9 | 2443*** | 1597.00 | 9 |
| Submerged in plates | A549 + THP-1 | 0 | 100.00 | 8.43 | 9 | 100.00 | 12.69 | 9 | 100.00 | 10.88 | 9 | 100.00 | 12.01 | 9 |
|  |  | 1 (µg/cm²) | 160.30 | 128.20 | 9 | 101.70 | 10.53 | 9 | 102.10 | 95.20 | 9 | 94.64 | 18.85 | 9 |
|  |  | 3 (µg/cm²) | 131.30 | 32.07 | 9 | 152.50 | 29.58 | 9 | 87.23 | 39.81 | 9 | 192.50 | 160.10 | 9 |
|  |  | 10 (µg/cm²) | 160.6* | 48.46 | 9 | 249.2** | 78.65 | 9 | 66.49 | 35.25 | 9 | 200.30 | 154.80 | 9 |
|  |  | 20 (µg/cm²) | 221.7*** | 39.83 | 9 | 371.6*** | 190.30 | 9 | 123.30 | 32.50 | 9 | 268.90 | 243.30 | 9 |
|  |  | LPS | 1559*** | 2208.00 | 8 | 2216*** | 1706.00 | 6 | 1675.00 | 1273.00 | 4 | 2406*** | 2849.00 | 9 |

**Table S4. IL-1β results expressed in percentage compared to control.**

|  |  |  | **NM105** | | | **NM101** | | | **NM100** | | | **NM212** | | |
| --- | --- | --- | --- | --- | --- | --- | --- | --- | --- | --- | --- | --- | --- | --- |
| Exposure method | Cell model | Dose | Mean | SD | n | Mean | SD | n | Mean | SD | n | Mean | SD | n |
| ALI. secretion in Basal side | A549 | 0 | 100.00 | 13.09 | 9 | 100.00 | 11.56 | 9 | 100.00 | 27.99 | 9 | 100.00 | 17.08 | 9 |
|  |  | 3 (µg/cm²) | 113.00 | 30.30 | 9 | 98.47 | 15.11 | 9 | 108.60 | 34.36 | 9 | 112.10 | 61.55 | 9 |
|  |  | LPS | 297.5*** | 145.50 | 6 | 237.3** | 34.86 | 6 | 209.9*** | 39.64 | 6 | 292.4** | 65.62 | 6 |
|  | A549 + THP-1 | 0 | 100.00 | 5.70 | 9 | 100.00 | 24.41 | 9 | 100.00 | 41.92 | 9 | 100.00 | 61.17 | 9 |
|  |  | 0.1 (µg/cm²) | 105.30 | 33.96 | 8 | 96.41 | 75.82 | 9 | 103.30 | 31.84 | 9 | 123.10 | 48.41 | 9 |
|  |  | LPS | 5342** | 2229.00 | 9 | 2334* | 2418.00 | 4 | 260.5* | 113.60 | 4 | 1262*** | 498.60 | 6 |
|  |  | 0 | 100.00 | 29.64 | 9 | 100.00 | 25.97 | 9 | 100.00 | 65.32 | 9 | 100.00 | 21.69 | 9 |
|  |  | 1 (µg/cm²) | 327.8* | 120.90 | 9 | 331** | 104.50 | 9 | 205.00 | 49.54 | 9 | 109.20 | 26.14 | 9 |
|  |  | LPS | 1436*** | 383.30 | 6 | 981.5*** | 522.60 | 6 | 1133*** | 230.60 | 6 | 1549** | 1168.00 | 6 |
|  |  | 0 | 100.00 | 20.65 | 9 | 100.00 | 24.11 | 9 | 100.00 | 27.50 | 9 | 100.00 | 38.07 | 9 |
|  |  | 3 (µg/cm²) | 668*** | 467.70 | 9 | 164.9* | 39.00 | 9 | 447.4* | 328.40 | 9 | 206.7* | 69.10 | 9 |
|  |  | LPS | 7606*** | 3019.00 | 5 | 1805*** | 518.30 | 6 | 4896*** | 1758.00 | 6 | 641.1*** | 97.49 | 6 |
| Submerged in inserts. secretion in Basal side | A549 + THP-1 | 0 | 100.00 | 11.11 | 9 | 100.00 | 9.97 | 9 | 100.00 | 8.02 | 9 | 100.00 | 17.09 | 9 |
|  |  | 1 (µg/cm²) | 163.20 | 60.43 | 9 | 113.70 | 16.96 | 9 | 124.40 | 20.95 | 6 | 91.86 | 16.34 | 9 |
|  |  | 3 (µg/cm²) | 209.10 | 59.40 | 9 | 120.00 | 15.53 | 9 | 116.10 | 21.70 | 9 | 108.10 | 37.09 | 9 |
|  |  | 10 (µg/cm²) | 591** | 305.40 | 9 | 229.1** | 89.85 | 9 | 142.50 | 39.95 | 9 | 128.70 | 37.00 | 9 |
|  |  | LPS | 1527*** | 399.1 | 9 | 1527*** | 399.1 | 9 | 1527*** | 399.1 | 9 | 1527*** | 399.10 | 9 |
| Submerged in inserts. secretion in Apical side | A549 + THP-1 | 0 | 100.00 | 10.70 | 9 | 100.00 | 6.54 | 9 | 100.00 | 13.62 | 9 | 100.00 | 14.28 | 9 |
|  |  | 1 (µg/cm²) | 181.80 | 67.35 | 9 | 129.30 | 13.38 | 9 | 111.80 | 25.17 | 6 | 102.70 | 22.66 | 9 |
|  |  | 3 (µg/cm²) | 279.30 | 55.65 | 9 | 169.1* | 23.74 | 9 | 123.80 | 21.45 | 9 | 96.16 | 19.28 | 9 |
|  |  | 10 (µg/cm²) | 1323*** | 751.50 | 9 | 458.5*** | 84.88 | 9 | 183.2** | 64.49 | 9 | 161.00 | 58.01 | 9 |
|  |  | LPS | 10225*** | 3998.00 | 9 | 10225*** | 3998.00 | 9 | 10225*** | 3998.00 | 9 | 10225*** | 3998.00 | 9 |
| Submerged in plates | A549 + THP-1 | 0 | 100.00 | 7.44 | 9 | 100.00 | 12.16 | 9 | 100.00 | 14.63 | 9 | 100.00 | 7.15 | 9 |
|  |  | 1 (µg/cm²) | 110.80 | 19.58 | 9 | 109.00 | 11.02 | 9 | 80.35 | 7.10 | 6 | 125.30 | 86.97 | 9 |
|  |  | 3 (µg/cm²) | 107.10 | 34.89 | 9 | 104.00 | 13.25 | 9 | 97.97 | 46.50 | 9 | 136.40 | 65.47 | 9 |
|  |  | 10 (µg/cm²) | 142.80 | 41.68 | 9 | 135.8* | 25.52 | 9 | 90.15 | 22.75 | 9 | 147.70 | 61.34 | 9 |
|  |  | 20 (µg/cm²) | 174.8*** | 32.65 | 9 | 152.5*** | 24.83 | 9 | 106.00 | 27.86 | 9 | 151.80 | 83.45 | 9 |
|  |  | LPS | 241.1** | 171.50 | 7 | 180.7*** | 14.04 | 8 | 137.00 | 45.49 | 4 | 204.9** | 97.46 | 9 |

**Table S5. IL-6 results expressed in percentage compared to control.**

|  |  |  | **NM105** | | | **NM101** | | | **NM100** | | | **NM212** | | |
| --- | --- | --- | --- | --- | --- | --- | --- | --- | --- | --- | --- | --- | --- | --- |
| Exposure method | Cell model | Dose | Mean | SD | n | Mean | SD | n | Mean | SD | n | Mean | SD | n |
| ALI. secretion in Basal side | A549 | 0 | 100.00 | 21.50 | 9 | 100.00 | 38.21 | 9 | 100.00 | 44.02 | 9 | 100.00 | 18.42 | 9 |
|  |  | 3 (µg/cm²) | 105.30 | 51.23 | 9 | 83.32 | 49.39 | 9 | 137.20 | 103.20 | 9 | 119.30 | 33.07 | 9 |
|  |  | LPS | 494** | 166.30 | 6 | 577.7** | 84.83 | 6 | 391.10 | 228.80 | 6 | 667.1*** | 93.70 | 6 |
|  | A549 + THP-1 | 0 | 100.00 | 18.65 | 9 | 100.00 | 12.32 | 9 | 100.00 | 49.47 | 9 | 100.00 | 31.65 | 9 |
|  |  | 0.1 (µg/cm²) | 104.70 | 44.52 | 8 | 98.17 | 54.11 | 9 | 88.04 | 48.59 | 9 | 108.10 | 25.13 | 9 |
|  |  | LPS | 1143*** | 412.20 | 9 | 84.17 | 8.55 | 4 | 44.33* | 6.91 | 4 | 15.42** | 1.66 | 6 |
|  |  | 0 | 100.00 | 35.19 | 9 | 100.00 | 8.20 | 9 | 100.00 | 49.45 | 9 | 100.00 | 9.15 | 9 |
|  |  | 1 (µg/cm²) | 245.5** | 64.38 | 9 | 67.08** | 23.60 | 9 | 171.10 | 32.91 | 9 | 111.80 | 20.60 | 9 |
|  |  | LPS | 485.7*** | 210.50 | 6 | 43.18*** | 17.77 | 6 | 141.5** | 15.04 | 4 | 20.87* | 5.72 | 6 |
|  |  | 0 | 100.00 | 21.11 | 9 | 100.00 | 5.80 | 9 | 100.00 | 18.28 | 9 | 100.00 | 9.69 | 9 |
|  |  | 3 (µg/cm²) | 303.1** | 125.10 | 9 | 121.1* | 14.54 | 9 | 254.4*** | 136.70 | 9 | 66.08* | 19.05 | 9 |
|  |  | LPS | 416.7* | 334.30 | 5 | 51.90 | 25.56 | 6 | 130.80 | 17.78 | 6 | 18.68*** | 4.55 | 6 |
| Submerged in inserts. secretion in Basal side | A549 + THP-1 | 0 | 100.00 | 10.01 | 9 | 100.00 | 5.65 | 9 | 100.00 | 7.85 | 9 | 100.00 | 12.68 | 9 |
|  |  | 1 (µg/cm²) | 142.20 | 41.50 | 9 | 115.90 | 16.35 | 9 | 103.10 | 17.10 | 9 | 99.63 | 6.45 | 9 |
|  |  | 3 (µg/cm²) | 191.4** | 34.43 | 9 | 127.4* | 11.11 | 9 | 98.64 | 16.78 | 9 | 100.50 | 13.16 | 9 |
|  |  | 10 (µg/cm²) | 283.8*** | 68.79 | 9 | 183*** | 41.30 | 9 | 126.50 | 37.59 | 9 | 141.0** | 25.56 | 9 |
|  |  | LPS | 143.1 | 44.19 | 9 | 143.1** | 44.19 | 9 | 143.1* | 44.19 | 9 | 143.1* | 44.19 | 9 |
| Submerged in inserts. secretion in Apical side | A549 + THP-1 | 0 | 100.00 | 8.75 | 9 | 100.00 | 4.51 | 9 | 100.00 | 6.39 | 9 | 100.00 | 7.89 | 9 |
|  |  | 1 (µg/cm²) | 134.60 | 25.78 | 9 | 128.90 | 18.12 | 9 | 102.00 | 11.80 | 9 | 98.89 | 8.25 | 9 |
|  |  | 3 (µg/cm²) | 159.9* | 29.80 | 9 | 144.8* | 9.73 | 9 | 107.00 | 6.13 | 9 | 97.60 | 12.60 | 9 |
|  |  | 10 (µg/cm²) | 141.60 | 85.69 | 9 | 167.2*** | 25.40 | 9 | 138.7** | 32.07 | 9 | 118.00 | 7.11 | 9 |
|  |  | LPS | 24.67 | 11.33 | 9 | 24.67 | 11.33 | 9 | 24.67 | 11.33 | 9 | 24.67** | 11.33 | 9 |
| Submerged in plates | A549 + THP-1 | 0 | 100.00 | 11.45 | 9 | 100.00 | 8.85 | 9 | 100.00 | 7.99 | 9 | 100.00 | 7.13 | 9 |
|  |  | 1 (µg/cm²) | 118.50 | 34.81 | 9 | 114.40 | 18.63 | 9 | 145.00 | 53.32 | 9 | 99.17 | 22.23 | 9 |
|  |  | 3 (µg/cm²) | 110.00 | 30.53 | 9 | 85.42 | 17.56 | 9 | 128.90 | 42.86 | 9 | 92.89 | 29.56 | 9 |
|  |  | 10 (µg/cm²) | 99.63 | 24.30 | 9 | 99.43 | 24.54 | 9 | 142.80 | 59.39 | 9 | 88.26 | 29.99 | 9 |
|  |  | 20 (µg/cm²) | 75.57 | 17.17 | 9 | 110.20 | 12.26 | 9 | 96.68 | 14.39 | 9 | 81.90 | 28.13 | 9 |
|  |  | LPS | 443.9** | 65.93 | 7 | 48.05** | 21.03 | 6 | 47.97 | 19.48 | 4 | 53.79* | 21.61 | 9 |

**Table S6. IL-8 results expressed in percentage compared to control.**

|  |  |  | **NM105** | | | **NM101** | | | **NM100** | | | **NM212** | | |
| --- | --- | --- | --- | --- | --- | --- | --- | --- | --- | --- | --- | --- | --- | --- |
| Exposure method | Cell model | Dose | Mean | SD | n | Mean | SD | n | Mean | SD | n | Mean | SD | n |
| ALI. secretion in Basal side | A549 | 0 | 100.00 | 8.49 | 9 | 100.00 | 11.80 | 9 | 100.00 | 23.90 | 9 | 100.00 | 16.81 | 9 |
|  |  | 3 (µg/cm²) | 108.50 | 21.70 | 9 | 97.08 | 9.27 | 9 | 101.60 | 29.30 | 9 | 115.20 | 26.82 | 9 |
|  |  | LPS | 205** | 36.91 | 5 | 168.1** | 20.08 | 6 | 184*** | 36.72 | 7 | 185.7*** | 19.55 | 6 |
|  | A549 + THP-1 | 0 | 100.00 | 7.66 | 9 | 100.00 | 13.40 | 9 | 100.00 | 6.99 | 9 | 100.00 | 30.08 | 9 |
|  |  | 0.1 (µg/cm²) | 100.40 | 16.54 | 8 | 90.21 | 30.02 | 9 | 91.64 | 25.91 | 9 | 96.62 | 18.83 | 9 |
|  |  | LPS | 756.9** | 150.90 | 9 | 212.80 | 101.60 | 4 | 182.70 | 71.25 | 4 | 277.2** | 40.91 | 6 |
|  |  | 0 | 100.00 | 28.03 | 9 | 100.00 | 24.80 | 9 | 100.00 | 45.98 | 9 | 100.00 | 11.60 | 9 |
|  |  | 1 (µg/cm²) | 254.1* | 87.29 | 9 | 186.9* | 32.64 | 9 | 150.40 | 30.39 | 9 | 114.10 | 18.34 | 9 |
|  |  | LPS | 2828*** | 2377.00 | 6 | 307.8*** | 58.22 | 6 | 194.8* | 72.72 | 6 | 164.2** | 61.84 | 6 |
|  |  | 0 | 100.00 | 13.84 | 9 | 100.00 | 10.80 | 9 | 100.00 | 18.24 | 9 | 100.00 | 15.84 | 9 |
|  |  | 3 (µg/cm²) | 308.4** | 181.20 | 9 | 143.9* | 40.83 | 9 | 251.80 | 147.90 | 9 | 89.87 | 15.76 | 9 |
|  |  | LPS | 1099*** | 694.60 | 5 | 392.3*** | 140.60 | 6 | 481.9*** | 87.95 | 6 | 227.6* | 53.88 | 6 |
| Submerged in inserts. secretion in Basal side | A549 + THP-1 | 0 | 100.00 | 9.59 | 9 | 100.00 | 12.33 | 9 | 100.00 | 5.75 | 9 | 100.00 | 14.48 | 9 |
|  |  | 1 (µg/cm²) | 127.00 | 30.56 | 9 | 105.40 | 17.55 | 9 | 103.20 | 11.33 | 9 | 114.00 | 24.36 | 8 |
|  |  | 3 (µg/cm²) | 167* | 29.14 | 9 | 131.70 | 45.21 | 9 | 96.86 | 14.54 | 9 | 103.80 | 10.46 | 9 |
|  |  | 10 (µg/cm²) | 264.8*** | 55.14 | 9 | 154.4** | 26.08 | 9 | 117.30 | 21.53 | 9 | 129.90 | 27.22 | 9 |
|  |  | LPS | 179.6** | 33.32 | 9 | 179.6*** | 33.32 | 9 | 179.6*** | 33.32 | 9 | 179.6*** | 33.32 | 9 |
| Submerged in inserts. secretion in Apical side | A549 + THP-1 | 0 | 100.00 | 5.88 | 9 | 100.00 | 5.13 | 9 | 100.00 | 14.82 | 9 | 100.00 | 9.31 | 9 |
|  |  | 1 (µg/cm²) | 139.40 | 28.89 | 9 | 126.90 | 25.34 | 9 | 95.95 | 14.61 | 9 | 106.00 | 21.58 | 8 |
|  |  | 3 (µg/cm²) | 173** | 31.59 | 9 | 152.2* | 15.84 | 9 | 106.00 | 17.87 | 9 | 91.59 | 16.73 | 9 |
|  |  | 10 (µg/cm²) | 172.6* | 65.73 | 9 | 197.8*** | 17.04 | 9 | 134.40 | 28.00 | 9 | 110.10 | 19.33 | 9 |
|  |  | LPS | 243*** | 44.67 | 9 | 243.0*** | 44.67 | 9 | 243*** | 44.67 | 9 | 243.0** | 44.67 | 9 |
| Submerged in plates | A549 + THP-1 | 0 | 100.00 | 8.21 | 9 | 100.00 | 6.81 | 9 | 100.00 | 5.37 | 9 | 100.00 | 10.00 | 9 |
|  |  | 1 (µg/cm²) | 121.40 | 29.52 | 9 | 99.91 | 4.20 | 9 | 102.50 | 34.63 | 9 | 100.00 | 12.41 | 8 |
|  |  | 3 (µg/cm²) | 109.40 | 39.40 | 9 | 113.00 | 9.44 | 9 | 117.90 | 59.98 | 9 | 138.70 | 63.93 | 9 |
|  |  | 10 (µg/cm²) | 150.90 | 48.71 | 9 | 147.7** | 24.38 | 9 | 86.54 | 26.00 | 9 | 139.00 | 50.13 | 9 |
|  |  | 20 (µg/cm²) | 188** | 39.25 | 9 | 183.6*** | 23.49 | 9 | 169.80 | 98.06 | 9 | 162.10 | 79.67 | 9 |
|  |  | LPS | 389.5*** | 182.20 | 6 | 1781*** | 815.80 | 6 | 450** | 75.01 | 4 | 2208*** | 1040.00 | 9 |

**Table S7. TNF-α results expressed in percentage compared to control.**

|  |  |  | **values in pg/mL** | | | | | | **Values in %/control** | | | | | |
| --- | --- | --- | --- | --- | --- | --- | --- | --- | --- | --- | --- | --- | --- | --- |
|  |  |  | Basal | | | Apical | | | Basal | | | Apical | | |
| Exposure method | Cell model | treatment | Mean | SD | n | Mean | SD | n | Mean | SD | n | Mean | SD | n |
| Submerged | A549 + THP-1 | 0 | 9.273 | 1.84 | 9 | 14.46 | 5.17 | 9 | 100 | 15.5 | 9 | 100 | 8.12 | 9 |
|  |  | LPS (20 µg/mL) | 81.77 | 54.9 | 9 | 445.7 | 425 | 9 | 826.7 | 454 | 9 | 3982 | 4137 | 9 |
| ALI | A549 + THP-1 | 0 | 36.3 | 17.7 | 9 | 61.06 | 20.7 | 9 | 100 | 34.1 | 9 | 100 | 23 | 9 |
|  |  | LPS (20 µg/mL) | 444.8 | 114 | 6 | 269.4 | 147 | 6 | 1357 | 615 | 6 | 415.9 | 154 | 6 |

**Table S8. IL-1β levels at the basal and apical sides after stimulation with LPS (20 µg/mL).**

|  |  |  | **values in pg/mL** | | | | | | **Values in %/control** | | | | | |
| --- | --- | --- | --- | --- | --- | --- | --- | --- | --- | --- | --- | --- | --- | --- |
|  |  |  | Basal | | | Apical | | | Basal | | | Apical | | |
| Exposure method | Cell model | treatment | Mean | SD | n | Mean | SD | n | Mean | SD | n | Mean | SD | n |
| Submerged | A549 + THP-1 | 0 | 39.96 | 15.7 | 9 | 98.38 | 79 | 9 | 100 | 17.1 | 9 | 100 | 13.9 | 9 |
|  |  | LPS (20 µg/mL) | 591.2 | 204 | 9 | 9267 | 5386 | 9 | 1527 | 399 | 9 | 16324 | 12978 | 9 |
| ALI | A549 + THP-1 | 0 | 138.1 | 36.4 | 9 | 635 | 274 | 9 | 100 | 23.5 | 9 | 100 | 22.2 | 9 |
|  |  | LPS (20 µg/mL) | 1059 | 636 | 6 | 1278 | 666 | 6 | 790.9 | 539 | 6 | 195.9 | 47.8 | 6 |

**Table S9. IL-6 levels at the basal and apical sides after stimulation with LPS (20 µg/mL).**

|  |  |  | **values in pg/mL** | | | | | | **Values in %/control** | | | | | |
| --- | --- | --- | --- | --- | --- | --- | --- | --- | --- | --- | --- | --- | --- | --- |
|  |  |  | Basal | | | Apical | | | Basal | | | Apical | | |
| Exposure method | Cell model | treatment | Mean | SD | n | Mean | SD | n | Mean | SD | n | Mean | SD | n |
| Submerged | A549 +  THP-1 | 0 | 15723 | 4471 | 9 | 25190 | 9546 | 9 | 100 | 12.68 | 9 | 100 | 7.887 | 9 |
|  |  | LPS (20 µg/mL) | 22184 | 7156 | 9 | 5172 | 1535 | 9 | 143.1 | 44.19 | 9 | 23.64 | 12.27 | 9 |
| ALI | A549 +  THP-1 | 0 | 39245 | 4436 | 9 | 14024 | 3257 | 9 | 100 | 8.2 | 9 | 100 | 21.67 | 9 |
|  |  | LPS (20 µg/mL) | 16978 | 6978 | 6 | 8660 | 3096 | 6 | 43.18 | 17.77 | 6 | 60.63 | 15.43 | 6 |

**Table S10. IL-8 levels at the basal and apical sides after stimulation with LPS (20 µg/mL).**

|  |  |  | **values in pg/mL** | | | | | | **Values in %/control** | | | | | |
| --- | --- | --- | --- | --- | --- | --- | --- | --- | --- | --- | --- | --- | --- | --- |
|  |  |  | Basal | | | Apical | | | Basal | | | Apical | | |
| Exposure method | Cell model | treatment | Mean | SD | n | Mean | SD | n | Mean | SD | n | Mean | SD | n |
| Submerged | A549 +  THP-1 | 0 | 57.44 | 23.11 | 9 | 75.4 | 9.314 | 9 | 100 | 14.48 | 9 | 100 | 5.51 | 9 |
|  |  | LPS (20 µg/mL) | 105.8 | 23.98 | 9 | 200.7 | 64.56 | 9 | 196.3 | 43.13 | 9 | 260.6 | 61.47 | 9 |
| ALI | A549 +  THP-1 | 0 | 98.43 | 24.49 | 9 | 110.1 | 70.82 | 9 | 100 | 23.12 | 9 | 100 | 24.82 | 9 |
|  |  | LPS (20 µg/mL) | 251.5 | 43.04 | 6 | 230.9 | 158 | 6 | 259 | 56.97 | 6 | 210 | 45.94 | 6 |

**Table S11. TNF-α levels at the basal and apical sides after stimulation with LPS (20 µg/mL).**

1. Taurozzi JS, Hackley VA, Wiesner MR. Ultrasonic dispersion of nanoparticles for environmental, health and safety assessment - issues and recommendations. Nanotoxicology. 2011;5(4):711-29.
